# Identifying Genes Associated with the Anticancer Activity of a Fluorinated Chalcone in Triple-Negative Breast Cancer Cells Using Bioinformatics Tools

**DOI:** 10.3390/ijms26083662

**Published:** 2025-04-12

**Authors:** Eduardo De la Cruz-Cano, José Ángel González-Díaz, Ivonne María Olivares-Corichi, Jorge Tonatiuh Ayala-Sumuano, José Alfredo Díaz-Gandarilla, Quirino Torres-Sauret, Violeta Larios-Serrato, Miguel Ángel Vilchis-Reyes, Carlos Javier López-Victorio, José Arnold González-Garrido, José Rubén García-Sánchez

**Affiliations:** 1 Laboratorio de Bioquímica y Biología Molecular, División Académica de Ciencias Básicas, Centro de Investigación de Ciencia y Tecnología Aplicada de Tabasco (CICTAT), Universidad Juárez Autónoma de Tabasco, Cunduacán C.P. 86690, Mexico; eduardocano239@gmail.com (E.D.l.C.-C.); jangel_gonzalez_1610@hotmail.com (J.Á.G.-D.); quirino.torres@ujat.mx (Q.T.-S.); miguel.vilchis@ujat.mx (M.Á.V.-R.); arnold.gonzalez@ujat.mx (J.A.G.-G.); 2 Laboratorio de Oncología Molecular y Estrés Oxidativo, Escuela Superior de Medicina, Instituto Politécnico Nacional, Ciudad de México C.P. 11340, Mexico; iolivares@ipn.mx; 3 IDIX Biotechnology, SA. de C.V. Querétaro, Querétaro C.P. 76235, Mexico; jsumuano@gmail.com; 4 Laboratorio de Análisis Clínicos, División Académica Multidisciplinaria de Comalcalco, Universidad Juárez Autónoma de Tabasco, Comalcalco C.P. 86650, Mexico; alfredo.diaz@ujat.mx; 5 Laboratorio de Biotecnología Genómica y Bioinformática, Escuela Nacional de Ciencias Biológicas, Instituto Politécnico Nacional, Ciudad de México C.P. 11340, Mexico; vlarios@ipn.mx

**Keywords:** DE genes, fluorinated chalcone, bioinformatic tools, TNBC

## Abstract

Fluorinated chalcones are molecules reported to possess potent anticancer properties against triple-negative breast cancer (TNBC) cells. However, their molecular mechanisms have not yet been fully explored. Using bioinformatics tools, we analyzed the transcriptomes of MDA-MB-231 cells treated with either a novel fluorinated chalcone (compound **3**) or a control in order to identify differentially expressed (DE) genes associated with its anticancer activity and determine the biological processes in which these genes are involved. A fluorinated chalcone was synthesized using the Claisen–Schmidt method. The transcriptome of MDA-MB-231 cells was then analyzed on an Illumina NextSeq500, and DE genes with significant changes in expression were identified using the DESeq2 v1.38.0 bioinformatics tool under the strict detection criteria of |log2FC| ≥  2 and adjusted *p* < 0.05. We identified 504 DE genes, which were enriched in terms related to “regulation of cell death”, “cation transport”, “response to topologically incorrect proteins”, and “response to unfolded proteins”. Surprisingly, these genes were involved in “the HSF1-dependent transactivation pathway” and “the attenuation phase pathway”. This bioinformatics-based study suggests that the tested fluorinated chalcone could influence HSF-1 silencing in addition to promoting the up-regulation of several genes involved in stress-induced apoptosis. Therefore, the tested compound could have enormous potential as a novel approach for TNBC treatment.

## 1. Introduction

Triple-negative breast cancer (TNBC) is one of the most aggressive malignancies worldwide, accounting for about 10–20% of all breast cancer cases [1]. Phenotypically, this condition is characterized by a lack of estrogen receptor (ER) and progesterone receptor (PR) expression as well as the absence of human epidermal growth factor receptor 2 (HER-2) amplification [1,2]. This is one of the main reasons why TNBC patients do not respond to hormonal therapies or anti-HER2 targeted treatment [2,3]. At present, the standard treatments for this condition are chemotherapy [4], PD-1/PD-L1 checkpoint inhibitors [5,6], and poly ADP-ribose polymerase (PARP) inhibitors [7,8]. Nevertheless, the majority of these treatments are ineffective and confer little benefit to patients suffering from this condition [4,9]. Therefore, there is a clear need for more effective, non-toxic compounds with good selectivity against cancer cells. In this context, chalcones [(1,3-diphenyl)-2-propen-1-ones] are organic molecules with simple structures consisting of two aromatic rings linked by three carbons in an α,β-unsaturated carbonyl system [10,11]. It has been shown that these compounds have anticancer activities and act by regulating important mechanisms such as the induction of apoptosis [12,13], the inhibition of cell growth [14,15], the inhibition of angiogenesis [16,17], and the suppression of the epithelial-to-mesenchymal transition [14], among others. It should be noted that, in the search to improve these properties, chalcones have been the object of fascinating bioisosteric replacements, with monovalent fluorine substitution being one of the most-studied for the following reasons: (a) fluorine-substituted chalcones have been reported to have greater metabolic stability than their non-fluorinated counterparts, which has been attributed to the high electronegativity of fluorine, resulting in chalcones with strongly stable C-F bonds [18,19]; (b) fluorine has been documented to act as a functional mimetic of carbonyl, carbinol, and nitrile moieties, significantly influencing the structural conformation of these compounds and their binding to target molecules [20]; (c) fluorine has been reported to exhibit greater lipophilicity compared to hydrogen, resulting in a higher permeability of these compounds in the cell membrane [21,22]; and (d) fluorine has been documented to reduce the basicity of chalcones, improving their bioavailability and consequently their anticancer activity [22,23]. In this context, although the anticancer roles of fluorinated chalcones have been extensively studied through different experimental assays—including molecular docking studies [24,25], gene expression studies [26,27], microarray assays [28], and so on—the nature of these approaches are limited in terms of understanding the possible molecular mechanisms of these compounds in the context of TNBC. In this regard, we hypothesized that fluorinated chalcones could induce the up-regulation of novel genes involved in the inhibition of cell migration, tumor progression, invasiveness, and metastasis, as well as the up-regulation of novel genes implicated in the induction of apoptosis in TNBC cells. For this reason, in this study, MDA-MB-231 cells were selected due to their highly aggressive phenotype and widespread use as a representative TNBC model [29,30]. Then, using a purely bioinformatics approach, we set out to analyze the transcriptome of these cells on a global scale—both those treated with a fluorinated chalcone and a control—in order to identify differentially expressed (DE) genes associated with the anticancer activity of this compound, as well as to identify the biological processes and molecular pathways in which these genes participate.

## 2. Results

### 2.1. Synthesis of (E)-3-(4-Fluorophenyl)-1-(2-pyrazinyl)-prop-2-en-1-one

The reaction was performed as described in Section 4. Upon completion, a solid was obtained, which was then washed with distilled water, crystallized with dichloromethane/hexane, and dried at 40 °C to obtain (*E*)-3-(4-fluorophenyl)-1-(pyrazin-2-yl)-prop-2-en-1-one (compound **3**), obtained as a green solid (86.7 mg, 67%), m. p. 118–120 °C. ^1^H NMR (600 MHz, CDCl_3_) δ 9.37 (d, *J* = 1.5 Hz, 1H), 8.77 (d, *J* = 2.4 Hz, 1H), 8.68 (dd, *J* = 2.4, 1.5 Hz, 1H), 8.10 (d, *J* = 16.0 Hz, 1H), 7.94 (d, *J* = 16.0 Hz, 1H), 7.71 (dd, *J* = 8.6, 5.5 Hz, 2H), 7.12 (t, *J* = 8.6 Hz, 2H). ^13^C NMR-DEPTQ (151 MHz, CDCl_3_) δ 188.4, 164.4 (d, *J* = 252.7 Hz), 148.5, 147.5, 144.9, 144.4, 143.4, 131.1 (d, *J* = 3.5 Hz), 130.9 (d, *J* = 8.5 Hz), 119.9 (d, *J* = 2.3 Hz), 116.2 (d, *J* = 21.8 Hz). The NMR spectra are provided in Supplementary File S1.

### 2.2. Fluorinated Chalcone Induces Morphological Changes Suggesting Apoptosis

**Figure 1:**
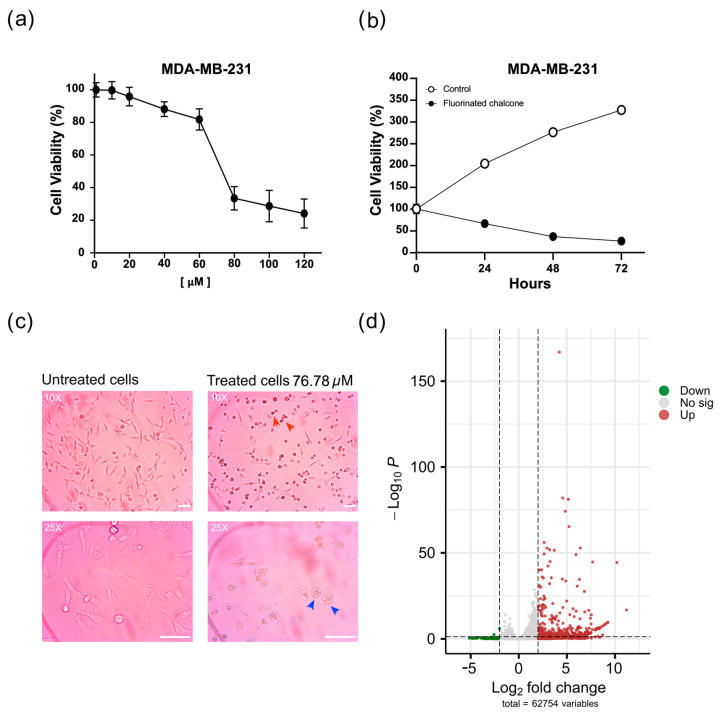
Effects of fluorinated chalcone (compound **3**) on MDA-MB-231 cell viability and distribution of the DE genes. (**a**) Cell viability of MDA-MB-231 cells treated with the indicated concentrations of 3 (0–120 μM) for 48 h. Data are presented as mean ± SD from triplicate data. (**b**) The anticancer effects of compound 3 at 24, 48, and 72 h. (**c**) Morphological changes in MDA-MB-231 cells treated with compound 3 suggestive of apoptosis, such as contraction and rounding of the cells (red arrows), as well as the formation of blisters on the cell membrane (blue arrows). Scale bars represent 100 μm. (**d**) Volcano plot showing the genes that were obtained in the differential expression analysis. The *y*-axis represents the –log10 adjusted *p*-values, indicating the level of significance of each gene, while the *x*-axis represents the log2 fold change, indicating the difference between the expression levels of each gene between both conditions. Red dots represent up-regulated genes, green dots show down-regulated genes, and gray dots indicate those genes that showed no significant changes.

To determine the optimal concentration of the fluorinated chalcone (compound **3**), we first evaluated the viability of MDA-MB-231 cells using the MTT assay. We identified that 76.78 ± 4.0 μM of compound **3** reached the IC_50_ in the treated cultures (Figure 1a). Second, to elucidate the anticancer effect of compound **3** over time, a follow-up experiment spanning 72 h was conducted. MDA-MB-231 cells were incubated with compound **3** at its IC_50_ concentration (76.78 μM) or without the fluorinated chalcone, and the effects were assessed at 24 h intervals (Figure 1b). For these observations, we examined the morphology of both the treated and control cells using light microscopy. Significantly, MDA-MB-231 cells exposed to compound **3** exhibited morphological and structural alterations suggestive of apoptosis-induced cell death [31,32]. These alterations included cell shrinkage, rounding, membrane blebbing, and cytoplasmic vacuolation, as depicted in Figure 1c.

### 2.3. Identifying DE Genes Associated with the Anticancer Activity of Fluorinated Chalcone

**Figure 2:**
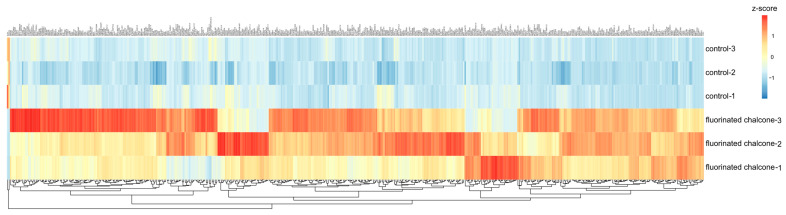
Heatmap of DE genes. The color scale represents log10 (i.e., normalized) expression values of DE genes. The horizontal axis represents the samples and the vertical axis represents DE genes, whose names are shown above. Red indicates up-regulated genes and blue represents down-regulated genes.

Using the DESeq2 v1.38.0 bioinformatics tool, 62,754 genes were obtained from the differential expression analysis (Figure 1d), of which 504 genes met the criteria for screening DE genes (i.e., |log2FC| >  2 and adj-*p* < 0.05). Specifically, 502 genes were found to be up-regulated (|log2FC| ≥  2; adj-*p* < 0.05), while only 2 DE genes were found to be down-regulated (|log2FC| ≤ −2; adj-*p* < 0.05). It is of great interest to highlight that, of the 502 up-regulated genes, approximately 55% were linked to ion transport and stress response pathways, underscoring the compound’s impact on cellular homeostasis. Among them, several members of the voltage-gated potassium channel (VGKC), voltage-gated calcium channel (VGCC), solute carrier (SLC), ATP-binding cassette (ABC) transporter, heat shock protein (HSP), and Kelch-like (KLHL) gene families were identified (Table 1). Additionally, a heatmap indicating all DE genes that were found to be regulated by compound **3** in MDA-MB-231 cells is shown in Figure 2.

### 2.4. Identifying the Biological Processes and Molecular Pathways of DE Genes

**Figure 3:**
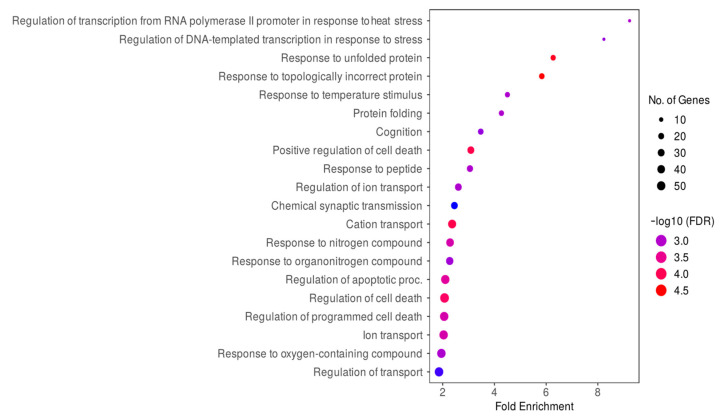
GO enrichment analysis representing the biological processes in which the DE genes are involved. The most significant processes are highlighted in red and the least significant in blue, based on -log10 (FDR) values. Larger dots on the graph indicate a greater number of genes involved. Abbreviation: FDR: False discovery rate.

To elucidate the biological processes and molecular pathways of the DE genes associated with the anticancer activity of compound **3** in MDA-MB-231 cells, an enrichment of GO terms and a pathway analysis were performed using the ShinyGO v0.80 and Reactome pathway Database v87, respectively. Regarding the enrichment of GO terms, we identified that these genes were significantly enriched in terms related to “regulation of cell death”, “cation transport”, “positive regulation of cell death”, “response to topologically incorrect proteins”, and “response to unfolded proteins” (Figure 3).

**Figure 4:**
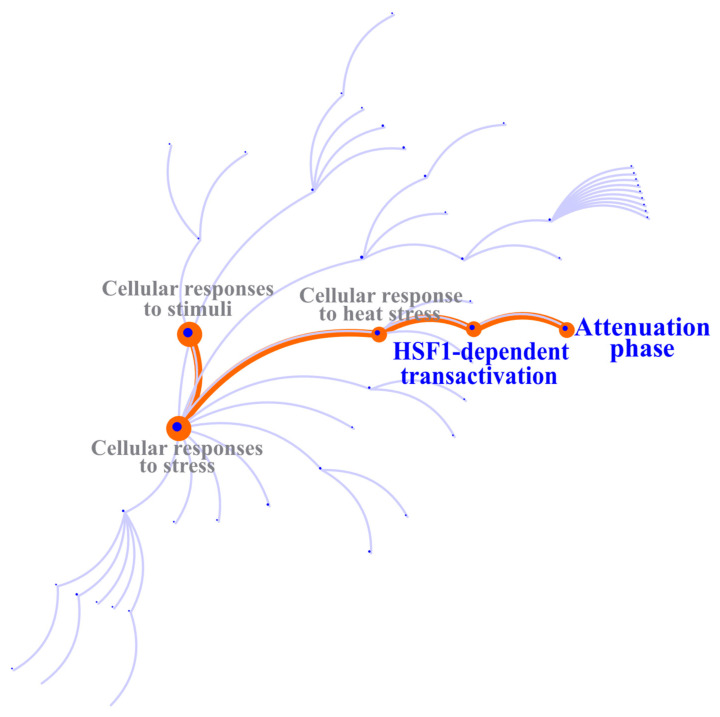
Representation of Reactome pathway analysis. The orange color denotes those pathways in which the DE genes were involved, while those that were found to be significantly represented by these genes are indicated in blue letters.

On the other hand, a total of 2698 pathways were explored to determine those in which the considered genes were involved. Interestingly, of all these pathways, only two reached the designated level of statistical significance (FDR  <  0.05): “the HSF1-dependent transactivation pathway” and “the attenuation phase pathway” (Table 2). An overview of the pathway analysis is presented in Figure 4, highlighting those that were stimulated by compound **3**.

### 2.5. Clusters of Genes Interact Synergistically to Inhibit Cell Proliferation of MDA-MB-231 Cells

**Figure 5:**
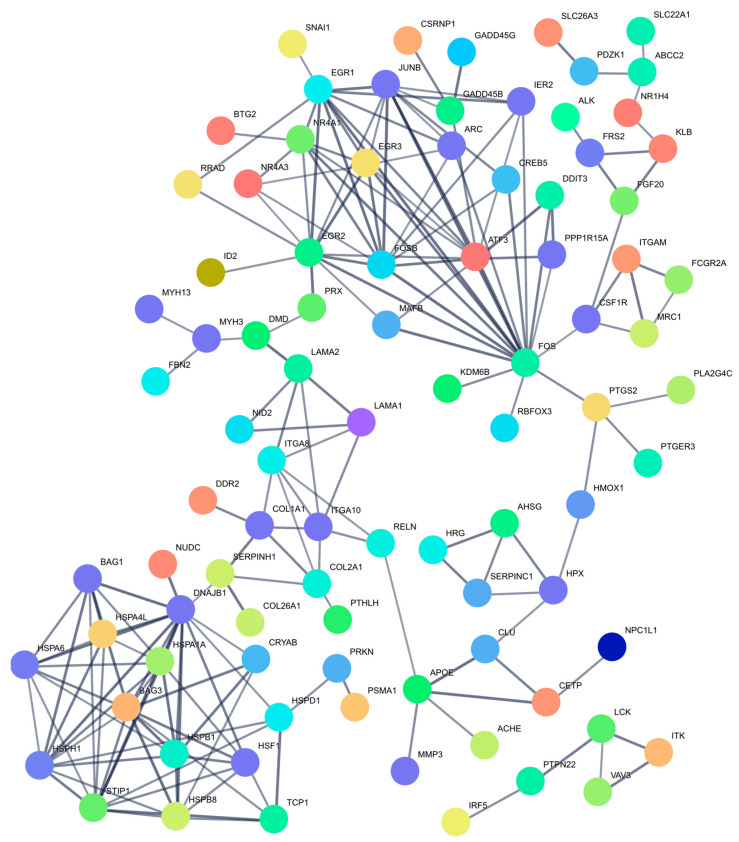
Protein–protein interaction network visualized using STRING. The nodes indicate proteins and the gray lines indicate the number of interactions. The saturation of lines represents the confidence scores of the functional associations. Disconnected nodes are hidden, and only those interactions with a confidence score of 0.7 are shown. Abbreviations: STRING: Search Tool for the Retrieval of Interacting Genes/Proteins.

**Figure 6:**
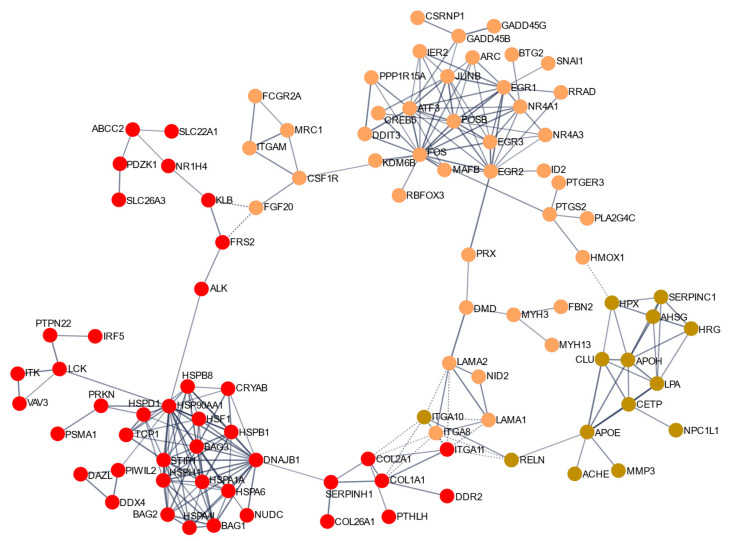
Application of the k-means algorithm for the identification of clusters in the STRING interaction network. Nodes represent proteins and edges indicate known interactions. The dotted lines indicate the connections between clusters. Colors differentiate the three obtained groups, highlighting functional modules within the network.

Regarding the statistics of the PPI network analysis obtained using the STRING bioinformatics tool, we identified a total of 90 nodes and 54 edges (Figure 5), with an average node degree of 0.997, a clustering coefficient of 0.226, a confidence value of 0.7, and an enrichment *p*-value of PPI < 1.0 × 10^−16^. Relevantly, we identified three important clusters. The first cluster comprised several heat shock proteins, including HSPA1A, HSPA4L, HSPA6, HSPH1, HSPB8, HSPB1, and CRYAB; HSP regulatory proteins, such as BAG1, BAG3, and STIP1; as well as the transcription factor HSF1. The second cluster consisted of several transcription factors, including FOS, MAFB, FOSB, JUNB, DDIT3, NR4A1, NR4A3, and ATF3; as well as proteins involved in cell cycle arrest and cell death, such as ID2, RRAD, EGR1, EGR2, EGR3, ARC, PPP1R15A, GADD45B, GADD45G, SNAI1, and BTG2. Finally, the third group consisted of genes APOH, LPA, HRG, SERPINC1, AHSG, HPX, CLU, APOE, CETP, NPC1L1, MMP3, ACHE, RELN, and ITGA10. Figure 6 shows these genes grouped into clusters.

## 3. Discussion

**Figure 7:**
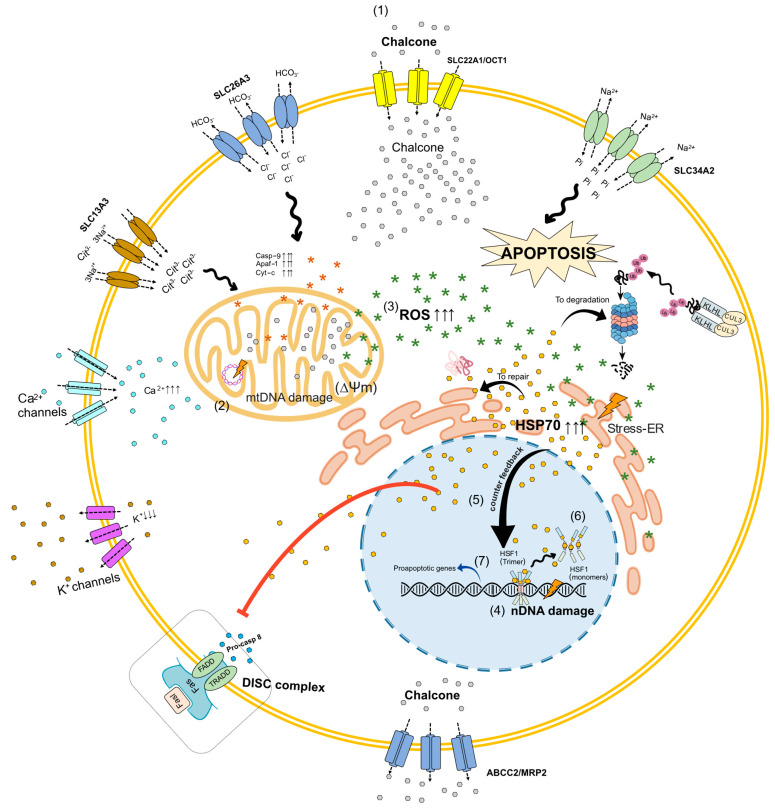
A possible apoptotic mechanism of the tested fluorinated chalcone in MDA-MB-231 cells. (1) Due to the physicochemical characteristics of the fluorinated chalcone, it could enter cells by passive diffusion or via specific cellular transporters such as the basolateral organic cation transporter (OCT-1). (2) Accumulation of the fluorinated chalcone at the intracellular level leads to alterations in nDNA and mtDNA and perturbations in the electron transport system, as well as alterations in the mitochondrial membrane potential (∆Ψm), which leads to an increase in the production of reactive oxygen species (ROS) and oxidative stress. (3) ROS trigger endoplasmic reticulum (ER) stress which, in turn, further enhances ROS production. (4) In response to cellular damage, several cytoprotective mechanisms are activated, including the expression of *ATF3* heat shock proteins (HSPs70), and *HSF1*, as well as the activation of other stress-inducible genes. (5) The overproduction of HSPs70, in turn, triggers a counter-feedback mechanism involving the binding of HSPs70 to the HSF1 trimers, which leads to dissociation from the promoter and its conversion to the inactive monomeric form. (6) As a result, the dissociation of HSF1 increases the susceptibility of MDA-MB-231 cells to stress-induced apoptosis. (7) Finally, several apoptosis-inducing genes are activated, including potassium channels, calcium channels, SLC co-transporters, and KLHL, among others. Abbreviations: ∆ψm: membrane potential; Apaf-1: apoptosis protease-activating factor-1; Cytc: cytochrome C; nDNA: nuclear deoxyribonucleic acid; DISC: death-inducing signaling complex; ER: endoplasmic reticulum; *HSF1*: Heat Shock Factor 1; HSP: heat shock protein; KLHL: Kelch-like proteins; mtDNA: mitochondrial deoxyribonucleic acid; ROS: reactive oxygen species; Ub: ubiquitin.

It has been reported that fluorinated chalcones have better anticancer activity than chalcones that do not contain fluorine [18,19]. However, to the best of our knowledge, no studies have focused on analyzing the anticancer activity of these compounds in TNBC cells on a global scale using a purely bioinformatics-based approach. Relevantly, we identified 504 DE genes (502 up- and 2 down-regulated) which, surprisingly, were involved in several biological processes, including the regulation of cell death, cation transport, the positive regulation of cell death, the response to topologically incorrect proteins, and the response to unfolded proteins. Therefore, to better understand the findings of our bioinformatics analysis, we propose some possible hypotheses, as follows: (1) *The fluorinated chalcone influences the ionic homeostasis of potassium and calcium, leading to apoptosis.* This argument is proposed given that several members of the VGKC and VGCC families were found to be up-regulated in our study. In this regard, the up-regulation of VGKC genes has been reported to increase K^+^ depletion, inducing DNA fragmentation and suppressing the metastatic phenotype [33,34,35]. It has also been reported that the activation of potassium channels could induce cell cycle arrest in breast cancer cells through the activation of senescence programs [36,37]. Interestingly, two genes from this family (*KCNJ15* and *KCNB1*) that were up-regulated by the fluorinated chalcone in this study have previously been reported to be down-regulated in several human cancers [38,39,40], which further strengthens this hypothesis regarding the role of these genes in the induction of apoptosis, the inhibition of cell proliferation, and migration. On the other hand, the up-regulation of VGCC genes has been reported to promote calcium apoptotic signaling, which has been correlated with a sustained elevation of intracellular Ca^2+^ concentrations, the overproduction of ROS, and alterations in the mitochondrial membrane potential, leading to cellular damage and apoptosis of cancer cells [41,42]. This could explain the up-regulation of the *CACNA1G* and *CACNA1E* genes found in our study, which surprisingly have also been found to be down-regulated in several solid tumors and cases of acute myelogenous leukemia [43,44,45]. (2) *The fluorinated chalcone induces an imbalance of essential substrates for cellular metabolism.* This hypothesis is proposed given that several members of the SLC and ABC families were found to be up-regulated in our bioinformatics analysis. Regarding the SLC gene family, it has been reported that it participates in the transport of molecules involved in cellular metabolism, including succinate, α-ketoglutarate, glutathione, endogenous monoamines, phosphates, and HCO_3_^−^, among others [46,47]. It is important to highlight that several of the SLC genes identified in our bioinformatics analysis have been previously reported to play important roles in apoptosis; for example, *SLC13A3* is a transporter of Krebs cycle intermediates, whose up-regulation leads to the increased uptake of succinate and α-ketoglutarate into the cell, inducing cellular senescence and exacerbating oxidative damage [48]. *SLC26A3* functions as an electroneutral Cl^−^/HCO_3_^−^ exchanger and, therefore, its up-regulation leads to imbalances in intracellular pH levels [49,50]. *SLC22A1* is a transporter of organic cations and monoamines, as well as anticancer drugs, whose down-regulation has been reported in hepatocellular carcinoma [51,52], breast carcinoma [53], pancreatic carcinoma [54], and cholangiocellular carcinoma [55]. Their exact mechanism is not known; however, their decrease is likely related to a decrease in the ability of cancer cells to take up these molecules, which could be a major contributor to tumor progression and a reduced response to chemotherapy. *SLC34A2* is a phosphate transporter, whose up-regulation has shown inhibitory effects on cell growth, migration, and invasiveness in lung cancer [56]; its apoptotic effect is likely related to phosphate re-uptake into the cell [57,58]. Regarding the ABC gene family, it has been reported that it also participates in the transport of a wide variety of metabolic substrates across intra- and extracellular membranes, including lipids, sterols, and drugs. Among the ABC genes up-regulated by the considered fluorinated chalcone, the *ABCA13* and *ABCC9* genes have been previously reported to play an important role in the induction of apoptosis. For instance, *ABCA13* is a lipid transporter whose up-regulation has been associated with a better response to chemotherapy in breast cancer, as well as with longer disease-free survival after chemotherapy in colorectal cancer patients [59,60]. *ABCC9* encodes the sulfonylurea receptor 2 (SUR2) protein, which constitutes a fundamental sub-unit of various ATP-sensitive potassium channels [61]. Down-regulation of this gene has been reported in several human cancers, including retinoblastoma [62], leukemia [63], prostate carcinoma [64], and breast carcinoma [65]. (3) *The fluorinated chalcone triggers cytoprotective mechanisms in response to cellular stress.* This hypothesis is proposed given that several stress-related genes were found to be up-regulated in this study. Among them, several members of the HSP gene family were observed, which encode highly conserved proteins involved in both stress and cellular damage. Their functions are heterogeneous, ranging from acting as molecular chaperones by participating in protein folding and repair, promoting the assembly/disassembly of multi-protein complexes, and degrading topologically incorrect proteins, as well as participating in the inhibition of apoptosis by reducing the release of cytochrome c and inhibiting the formation of death-inducing signaling complex (DISC) [66,67]. The HSP genes found to be up-regulated in this study included *HSPA1A*, *HSPA4L*, *HSPA6*, *HSPA7*, *HSPB1*, *HSPB8*, *HSPD1*, and *HSPH1*, which have also been reported to play key roles in the nuclear transport of proteins involved in DNA repair as well as correcting the folding process of proteins involved in DNA repair [68,69,70]. This finding was also confirmed by the up-regulation of *ATF3*, which is a stress-induced transcription factor that plays vital roles in modulating metabolism, immunity, and oncogenesis, acting as a regulatory hub of the cellular adaptive response [71]. Robust evidence has indicated that *ATF3* is rarely detectable under basal conditions; however, its expression can be induced immediately upon the exposure of cells to several stimuli, such as anticancer compounds, toxic chemicals, DNA damage, genotoxic agents, oxidative stress, and oncogenic factors, all of which point to cell cycle arrest and apoptosis [71,72]. Interestingly, this argument is also consistent with several expression studies that have analyzed these genes in experimental conditions similar to ours. For example, Oh et al. [73] found that chalcones can induce the overexpression of HSP70 in MDA-MB-231 cells, suggesting that these compounds may attenuate the migratory and invasive capacity of these cells [73]. Mai et al. [74] and Silva et al. [75], by biological assays, also found that these compounds can induce the up-regulation of not only HSP70 but also HSP60 and ATF3 in cancer cells [73,74]. This indicates that chalcones could trigger stress-induced apoptosis in cancer cells. (4) *The fluorinated chalcone promotes the degradation of proteins involved in cell proliferation and metastasis via the proteasome system.* This is based on the fact that our bioinformatics analysis identified several up-regulated KLHL genes, which have been reported to play important roles in ubiquitination processes by interacting as substrates in the Cullin3 (Cul3) ubiquitin ligase complexes to promote the degradation of proteins involved in tumor progression [76,77]. Importantly, mutations causing the loss of function and aberrant methylation of these genes have been reported to facilitate the metastasis of cancer cells [77,78]. Among the genes belonging to this family, *KLHL25*, *KLHL3*, and *KLHL6* were found to be up-regulated by the fluorinated chalcone; curiously, these genes have been described to inhibit cell migration and invasiveness in lung carcinoma [76], diffuse large B-cell lymphoma [79], and chronic lymphocytic leukemia [80]. (5) *The fluorinated chalcone triggers chemoresistance mechanisms in MDA-MB-231 cells.* This hypothesis is proposed given that, in our bioinformatics analysis, *ABCC2* was found to be up-regulated. *ABCC2* is one of the best-known genes that has been found to play an important role in cellular chemoprotection and resistance against various anticancer drugs (e.g., doxorubicin, methotrexate, vincristine, vinblastine, paclitaxel, docetaxel, etoposide, mitoxantrone, and cisplatin), as it encodes multi-drug resistance protein 2 (MRP2) [81,82]. Other genes found to be up-regulated in our study that support this assumption were *SLC1A3*, *SLC16A1*, *LINC00426*, *LINC01234*, *LINC02577*, *MAFB*, *APOE*, *MMP3*, *APOH*, and *CETP*, which have been associated with increased chemoresistance in pancreatic carcinoma [83,84], osteosarcoma [85], breast carcinoma [86], multiple myeloma [87], and hepatocellular carcinoma [88]. While this may be intriguing from a therapeutic point of view, given that chemoresistance is one of the most critical mechanisms affecting the efficacy of anticancer compounds [89], this should not be surprising given that it is well-known that cancer cells often express protein systems that act as efflux pumps to eliminate bioactive compounds that are lethal to them, reducing their intracellular concentration and conferring multidrug resistance (MDR) [89,90]. Therefore, molecular studies addressing the anticancer activities of fluorinated chalcones (including the one tested here) in synergy with efflux pump inhibitors (including *ABCC2*/MRP2 inhibitors) are needed. Furthermore, a pathway analysis was carried out to identify in which pathways these genes are involved. Interestingly, according to the FDR values, the most significant pathways in which these genes were enriched were the HSF1-dependent transactivation pathway and the attenuation phase pathway. In this context, Heat Shock Factor 1 (*HSF1*) is a master transcriptional regulator of heat shock responsive (HSR) signaling, forming part of the most significant cellular protective mechanisms [91,92]. The structure of the protein encoded by *HSF1* is composed of five parts: the N-terminal DNA binding domain (DBD), the leucine zipper 1-3 (LZ1-3), the regulatory domain (RD), leucine zipper 4 (LZ4), and the C-terminal transactivation domain (TAD) [93,94]. It is of great interest to mention that, in the *HSF1*-dependent transactivation pathway, TAD is a fundamental component for the survival of breast cancer cells once they have been subjected to conditions of stress or cell death, as it drives transcriptional programs of non-canonical genes involved in multiple aspects of tumorigenesis, such as apoptosis inhibition, reprogramming of cell metabolism, angiogenesis, adjustment of the tumor microenvironment, proliferation, cell migration, and metastasis [92,95]. To the contrary, the attenuation phase pathway involves various mechanisms leading to *HSF1* silencing, including the release of HSF1 trimers from heat shock elements (HSEs) as well as the dissociation of HSF1 trimers in monomers (their inactive form) [96]. This suggests that the fluorinated chalcone could play an important role in HSF1 silencing, further sensitizing these cells to stress-induced apoptosis. This argument is supported by the fact that *HSF1* silencing occurs when large amounts of HSPs70 are expressed to saturate the hydrophobic regions of damaged proteins as a consequence of cellular stress [95], which is consistent not only with our functional analysis findings but also with the PPI network, in which several proteins of the HSP70 family (as well as HSP regulatory proteins) were observed to interact with *HSF1*. Curiously, the up-regulation of these proteins has also been reported to trigger a counter-feedback mechanism that involves the binding of HSPs to the HSF1 trimer, leading to the dissociation of the promoter and its conversion to the inactive monomeric form [95]. Additionally, it is also worth highlighting that our PPI network analysis identified several interactions between ATF3 and important transcriptional factors, which have been reported to contribute to the induction of apoptosis under conditions of cellular stress; for instance, it has been reported that the up-regulation of *ATF3* together with *FOS*, *JUN*, *EGR1*, *EGR3*, *ARC*, and *NR4A1* can improve the sensitivity of colon cancer cells to anticancer compounds, suggesting that these genes cooperate strongly under conditions of cellular stress via a coordinated transcriptional network to overcome chemoresistance and induce apoptosis [97,98]. Other studies have also indicated that stress-induced ATF3 can bind to the promoter regions of *GADD45* family members to activate them, while cancer-associated ATF3 can repress them [99,100]. Furthermore, the up-regulation of DDIT3 (an important ER stress-inducible protein) has been reported to be dependent on ATF3 activation, as it induces apoptotic transcriptional programs through the ATF4–ATF3–DDIT3–TNFRSF10B signaling axis in human lung carcinoma [101]. A possible mechanism summarizing the findings of our bioinformatics analysis is proposed in Figure 7.

Finally, to the best of our knowledge, this study is the first bioinformatics-based study that analyzes the anticancer activity of a fluorinated chalcone on a global scale and which also proposes a possible mechanism of such a compound in TNBC cells using bioinformatics tools. In this way, new insights into the potential molecular mechanisms of these compounds in the biological context of this condition are provided. Nevertheless, given the nature of our study—in which a purely bioinformatics approach was employed for the identification of these genes—this opens new opportunities for future research, allowing for the exploration of new genes that could be of great relevance both in the therapeutic sense and for the prognosis of TNBC. It should be noted that this study has some limitations inherent to its methodological design and computational approach. For example, as this study was not carried out on other cell lines, our results and their interpretation are limited to a breast cancer-specific type. Furthermore, given that our bioinformatics analysis identified only those genes with biologically significant changes in their expression using one of the more stringent criteria considered in computational analyses (i.e., |log2FC| ≥  2; adj-*p* < 0.05), it is likely that several genes with relatively small changes and that could be involved in the regulation of these DE genes have been discriminated. Finally, while our bioinformatics analysis offers a comprehensive view of gene expression, we are not unaware that the functional validation of key DE genes (e.g., via qPCR or protein assays) is required to confirm their roles.

## 4. Materials and Methods

### 4.1. Chemistry

#### 4.1.1. Equipment and Experimental Conditions

The reagents used in this study were purchased from Sigma-Aldrich and were not further purified. Melting points were determined on a Fisher–Johns apparatus and were uncorrected. For thin-layer chromatography (TLC) analysis, silica-gel coated aluminum plates 60 F_254_ (Merck, Toluca, México) were used, and visualization was achieved via UV quenching (254 nm). Solvents for column chromatography purification were purchased as technical-grade solvents and were purified before use. Column chromatography was performed using 230–400 mesh silica (Merck). The ^1^H-NMR and ^13^C-NMR-DEPTQ spectra were recorded at 25 °C on a Bruker Ascend™ 600 spectrometer (Billerica, MA, USA). Chemical shifts are expressed as δ (ppm) values relative to TMS as internal standard, and coupling constants (*J*) are given in Hertz. Spectra were recorded in CDCl_3_. Multiplicities are denoted as s (singlet), d (doublet), dd (doublet double), dt (triplet double), or t (triplet).

#### 4.1.2. Synthesis of (E)-3-(4-Fluorophenyl)-1-(2-pyrazinyl)-prop-2-en-1-one (Fluorinated Chalcone)

**Scheme 1:**
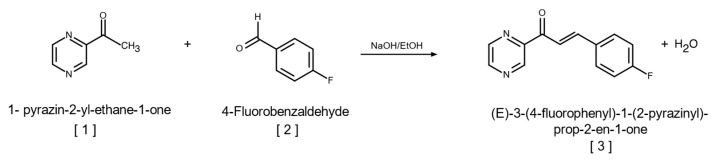
Reaction procedure for the synthesis of the fluorinated chalcone (compound **3**). The name of this compound is given according to the IUPAC. Abbreviations: EtOH: ethanol; IUPAC: International Union of Pure and Applied Chemistry; NaOH: sodium hydroxide.

The fluorinated chalcone (compound **3**) was synthesized via classical Claisen–Schmidt condensation based on a previously published article [102]. An equimolar mixture of 1-pyrazin-2-yl-ethane-1-one (61.1 mg, 0.5 mmol) and 4-fluorobenzaldehyde (61.1 mg, 0.5 mmol) was dissolved in 2.5 mL ethanol. Then, 1.5 mL of aqueous 1.0 M sodium hydroxide solution (0.5 mmol) was added dropwise. The resulting mixture was stirred vigorously at 35 °C for 20 h. After this time, the reaction was stopped and checked via TLC (hexane–ethyl acetate 9:1). The solid formed was then allowed to cool for 20 h and neutralized by adding 1 N hydrochloric acid, leading to formation of a precipitate. Next, the obtained precipitate was washed with distilled water, dried at 40 °C, and purified by column chromatography (hexane–ethyl acetate 9:1) to obtain the pure compound. The procedure for the synthesis of compound **3** is summarized in Scheme 1.

### 4.2. Cell Culture and Cell Viability Assay

MDA-MB-231 cells (American Type Culture Collection, Rockville, MD, USA) were used as a TNBC model, which were cultured in plates with Dulbecco’s Modified Eagle Medium (DMEM) supplemented with 10% fetal bovine serum (FBS) (Life Technologies, Carlsbad, CA, USA) and 100 µg/mL penicillin–streptomycin (Life Technologies, Carlsbad, CA, USA), and maintained in a humidified atmosphere at 37 °C with 5% CO_2_. When the cells reached 80% confluence, a solution of EDTA (1 mM in PBS) was used to detach them from the plates, following which they were sub-cultured (1 × 10^4^ cells/well) in 96-well culture plates and incubated for 24 h before treatment. Different concentrations of compound 3 (0–120 μM) were then prepared and added to the culture medium to treat the cells for 48 h. DMSO-treated cells were used as a negative control. After treatment, the 3-(4,5-dimethylthiazol-2-yl)-2,5-diphenyl tetrazolium bromide (MTT) assay was used to measure the viability of the MDA-MB-231 cells. Briefly, this assay was carried out by replacing the culture medium in each well with 100 μL of MTT (Sigma, St. Louis, MO, USA) (1 mg/mL in clear culture medium), followed by incubation for 1 h at 37 °C under 5% CO_2_. Subsequently, the medium was discarded and 100 μL of DMSO was applied to each well to dissolve the formazan crystals. Absorbance at 540 nm was measured with an iMarkTM Microplate Absorbance Reader (Bio-Rad Laboratories, Hercules, CA, USA). Finally, the half maximal inhibitory concentration (IC_50_) value was determined from three experiments by plotting a dose–response curve using the GraphPad Prism software v6.0.

#### Treatment of MDA-MB-231 Cells with the IC_50_

To investigate the effects of the developed compound, MDA-MB-231 cells were seeded in a 6-well cell culture plate at 1 × 10^4^ cells/well and allowed to attach for 24 h. Cells were then incubated with compound 3 at the determined IC_50_ value for 24 h (i.e., that previously obtained from the MTT assay). At 24 h of incubation time, the cells were detached with 1 mM EDTA, washed with PBS, and spun down by centrifugation (2000 rpm for 5 min), in order to collect the pellet prior to the corresponding assay. Morphological changes between treated and untreated groups were visualized using a TCM 400 inverted microscope (Labomed, Fremont, CA, USA) whose captured images were dimensionally estimated using ImageJ v1.53 software [103], and from which scale bars were generated for each image.

### 4.3. Library Preparation and RNA-Seq

After treatment at the IC_50_ value, total RNA was extracted from MDA-MB-231 cells according to a standard TRIZOL RNA isolation protocol (Life Technologies^®^, Inc., Grand Island, NY, USA). Nevertheless, to ensure good quality for sequencing, the total RNA was purified, according to the manufacturer’s protocol, with a PureLink™ RNA Mini Kit (Invitrogen, Santa Clara, CA, USA). The integrity and concentration of the RNA samples were determined through 2% agarose gel electrophoresis (visualized by ethidium bromide staining) and a Nanodrop 2000c spectrophotometer (Thermo Fisher Scientific, Waltham, MA, USA), respectively. The RNA Integrity Number (RIN) parameter was calculated using an Agilent 2100 Bioanalyzer system (Agilent Technologies, Santa Clara, CA, USA), such that only high-quality RNA (RIN ≥ 8.0) was selected for the construction of cDNA libraries. Next, the cDNA libraries were prepared using the TruSeq^®^ RNA sample preparation kit (Illumina, San Diego, CA, USA), according to the manufacturer’s protocol; once prepared, they had an average size of 300 bp. It is important to note that the libraries were validated before high-throughput sequencing using quantitative PCR (qPCR) and subsequently amplified in flow cells. Finally, the standard Illumina RNA-seq protocol was performed, according to the manufacturer’s instructions, in order to generate transcription profiles of the samples using sequencing by synthesis chemistry on the NextSeq500 equipment (Illumina, San Diego, CA, USA).

### 4.4. Bioinformatics Analysis

#### 4.4.1. Quality Control, Transcriptome Assembly, and Mapping

After sequencing, fastq.gz files containing the original RNA-seq reads were generated. FastQC v0.11.2 was then used to access the quality score distribution of these reads, such that low-quality reads (Phred score ≤ 15) could be removed using Trimmomatic v.0.039 [104]. Next, the remaining reads (i.e., those scored as good quality) were aligned to the human reference genome *GRCh38.p14* (https://www.ensembl.org/Homo_sapiens/Info/Index, accessed on 15 January 2024) using STAR v2.7.10b—a fast and sensitive spliced alignment software for mapping RNA-seq reads [105]. Finally, FeatureCounts v2.0.1 was used to count the reads by gene [106]. Importantly, the sequence data that support the findings of this study have been deposited in the National Center for Biotechnology Information (NCBI) with the primary accession code “PRJNA1135143”.

#### 4.4.2. Identification of Differentially Expressed (DE) Genes

Identification of DE genes was performed using the DESeq2 v1.38.0 package [107]. This bioinformatics tool provides methods for the analysis of high-throughput RNA-seq data to identify DE genes between different experimental conditions through the application of negative binomial distribution models [107,108]. For this purpose, the magnitude (log2 fold change) and significance (adjusted *p*-value) of differential expression between conditions (treated cells vs. untreated cells) were calculated, whereby those genes that met the strict detection conditions (i.e., |log2FC| ≥  2 and adjusted *p* < 0.05) were defined as differentially expressed. Here, it is important to emphasize that these thresholds were chosen to prioritize biologically significant expression changes while maintaining statistical rigor.

#### 4.4.3. Functional Analysis and Construction of a Protein–Protein Interaction (PPI) Network

To identify the biological processes in which the identified genes participate, an enrichment of gene ontology (GO) terms was carried out using the ShinyGO v0.80 package (http://bioinformatics.sdstate.edu/go/, accessed on 19 January 2024), taking a false discovery rate (FDR) ≤ 0.05 as a criterion. This tool allows for in-depth analysis of gene sets, with graphical visualization of enrichment as well as gene features, including hierarchical clustering trees, networks summarizing overlapping terms/pathways, and enriched promoter motifs, as well as plots of gene characteristics based on annotation from Ensembl [109]. On the other hand, in order to determine the pathways in which these genes were involved, the Reactome pathway database v87 was used, accessed on 22 January 2024. This bioinformatics tool is a free and open-source database of molecular pathways, reactions, and processes that allows for the analysis, interpretation, and visualization of the mechanisms of different biological entities (i.e., proteins, genes, and macromolecular complexes) involved in biological pathways [110]. The calculated *p*-values were corrected by taking FDR ≤ 0.05 as the threshold. Therefore, those pathways that met this condition were defined as significantly enriched. Furthermore, to better understand the biological interactions of these genes, a PPI network was constructed from DE genes using the Search Tool for Retrieval of Interacting Genes/Proteins (STRING) database (http://string-db.org/, accessed on 26 January 2024), which determines both direct (physical) and indirect (functional) associations between proteins [111]. Additionally, to reinforce the findings of our network analysis, we used the STRING’s k-means algorithm to group proteins into clusters based on the structure of the interaction network. The main criteria for clustering were as follows: *connectivity similarity*, which consists of grouping those proteins with similar interaction patterns; *interaction scores*, in which STRING assigns scores to interactions based on experimental evidence, co-expression, the literature, etc.; *feature space representation*, which converts the network into a vector space (e.g., using embeddings such as DeepWalk or adjacency matrices); *intra-cluster variance minimization*, in which k-means groups proteins by minimizing the difference between them within the same cluster and maximizing the difference with other clusters [111].

### 4.5. Statistical Analysis

The GraphPad Prism software v8.0.1 and R Studio software v3.4.3 were used to visualize the results of cell viability and conduct the differential expression analysis, respectively. For comparisons between both conditions, Student’s t-test of variance was performed. *p* < 0.05 was considered to indicate statistical significance.

## 5. Conclusions

In conclusion, using a purely bioinformatics-based approach, we identified several DE genes associated with the anticancer activity of the tested fluorinated chalcone in TNBC cells. The DE genes were involved in important biological processes such as cation transport, regulation of cell death, topologically incorrect protein response, and the response to unfolded proteins, among others. Likewise, the results of this study suggest that the fluorinated chalcone could influence HSF-1 silencing in addition to promoting the up-regulation of several genes involved in stress-induced apoptosis (e.g., *HSPs70*, *ATF3*, *FOS*, *JUN*, *EGR1*, *EGR3*, *ARC*, and *NR4A1*, among others). Therefore, the considered compound could have enormous potential for the treatment of TNBC and could serve as a possible therapeutic strategy for this condition. The findings presented here also provide novel molecular targets that should be explored in depth and could also be of great clinical relevance in TNBC. Additionally, the methodology and results presented in this bioinformatics analysis pave the way for more refined approaches, including biological assays that could allow us to better understand the molecular mechanisms of chalcones in the context of TNBC.

## Figures and Tables

**Table 1:** DE genes grouped by family.

Symbol	Gene Name	log2FC	*p*-Value	*p*-Adjusted
**Members of the VGKC family**				
*KCNB1*	Potassium voltage-gated channel subfamily B member 1	5.0077	0.0034	0.0456
*KCNH3*	Potassium voltage-gated channel subfamily H member 3	5.7809	2.19 × 10^−5^	0.0008
*KCNJ15*	Potassium inwardly rectifying channel subfamily J member 15	3.6421	0.0023	0.0337
*KCNN1*	Potassium calcium-activated channel subfamily N member 1	5.8723	1.74 × 10^−5^	0.0006
**Members of the VGCC family**				
*CACNA1E*	Calcium voltage-gated channel subunit alpha1 E	7.0921	7.82 × 10^−5^	0.0024
*CACNA1G*	Calcium voltage-gated channel subunit alpha1 G	6.3592	1.31 × 10^−14^	2.07 × 10^−12^
**Members of the SLC family**				
*SLC13A3*	Solute carrier family 13 member 3	5.5741	5.71 × 10^−6^	0.0002
*SLC16A1*	Solute carrier family 16 member 1	5.8161	0.0001	0.0045
*SLC17A8*	Solute carrier family 17 member 8	6.6355	0.0012	0.0220
*SLC22A1*	Solute carrier family 22 member 1	4.0406	0.0031	0.0430
*SLC22A20P*	Solute carrier family 22 member 20, pseudogene	3.5557	0.0025	0.0368
*SLC26A3*	Solute carrier family 26 member 3	5.3321	0.0011	0.0198
*SLC34A2*	Solute carrier family 34 member 2	5.8169	0.0016	0.0268
*SLC5A5*	Solute carrier family 5 member 5	6.1385	2.52 × 10^−8^	1.92 × 10^−6^
*SLC6A13*	Solute carrier family 6 member 13	6.2167	0.0014	0.0238
*SLC6A4*	Solute carrier family 6 member 4	5.0679	0.0037	0.0487
**Members of the ABC family**				
*ABCA13*	ATP-binding cassette subfamily A member 13	5.6202	0.0033	0.0447
*ABCC2*	ATP-binding cassette subfamily C member 2	2.1551	1.39 × 10^−7^	9.08 × 10^−6^
*ABCC9*	ATP-binding cassette subfamily C member 9	5.0750	3.47 × 10^−6^	0.0001
**Members of the HSP family**				
*HSPA1A*	Heat shock protein family A (Hsp70) member 1A	8.0281	1.44 × 10^−8^	1.16 × 10^−6^
*HSPA4L*	Heat shock protein family A (Hsp70) member 4 like	2.1221	9.57 × 10^−23^	3.57 × 10^−20^
*HSPA6*	Heat shock protein family A (Hsp70) member 6	10.1994	3.68 × 10^−48^	4.39 × 10^−45^
*HSPA7*	Heat shock protein family A (Hsp70) member 7 (pseudogene)	9.2484	2.1 × 10^−12^	2.77 × 10^−10^
*HSPB1*	Heat shock protein family B (small) member 1	2.5661	9.23 × 10^−4^	0.0170
*HSPB8*	Heat shock protein family B (small) member 8	3.3302	1.11 × 10^−6^	5.99 × 10^−5^
*HSPD1*	Heat shock protein family D (Hsp60) member 1	2.1816	1.61 × 10^−34^	1.11 × 10^−31^
**Members of the KLHL family**				
*KLHL25*	Kelch-like family member 25	2.0169	2.92 × 10^−6^	0.0001
*KLHL3*	Kelch-like family member 3	3.0216	4.1 × 10^−5^	0.0014
*KLHL6*	Kelch-like family member 6	6.1169	0.0021	0.0328

Abbreviations: ABC: ATP-binding cassette; HSP: heat shock protein; VGCC: voltage-gated calcium channel; VGKC: voltage-gated potassium channel; KLHL: Kelch-like; SLC: solute carrier. For a complete description of all the DE genes, see Supplementary File S2.

**Table 2:** Enriched pathways identified by Reactome among DE genes.

Identifier	Pathway *	Genes Found	Ratio	*p*-Value *	FDR *
R-HSA-3371571	HSF1-dependent transactivation	11/59	0.003	3.63 × 10^−7^	2.96 × 10^−4^
R-HSA-3371568	Attenuation phase	9/47	0.002	3.37 × 10^−6^	0.001
R-HSA-3371556	Cellular response to heat stress	16/305	0.013	0.004	0.977
R-HSA-3371453	Regulation of HSF1-mediated heat shock response	14/260	0.011	0.006	0.977
R-HSA-3371511	HSF1 activation	7/99	0.004	0.012	0.977
R-HSA-5083635	Defective B3GALTL causes PPs	4/39	0.002	0.016	0.977
R-HSA-8866906	TFAP2 (AP-2) family regulates transcription of other transcription factors	2/8	3.49 × 10^−4^	0.017	0.977
R-HSA-5173214	O-glycosylation of TSR domain-containing proteins	4/41	0.002	0.019	0.977
R-HSA-383280	Nuclear Receptor transcription pathway	7/113	0.005	0.023	0.977
R-HSA-9700649	Drug resistance of ALK mutants	1/1	4.37 × 10^−5^	0.024	0.977
R-HSA-9717301	NVP-TAE684-resistant ALK mutants	1/1	4.37 × 10^−5^	0.024	0.977
R-HSA-9717323	Ceritinib-resistant ALK mutants	1/1	4.37 × 10^−5^	0.024	0.977
R-HSA-9717329	Lorlatinib-resistant ALK mutants	1/1	4.37 × 10^−5^	0.024	0.977
R-HSA-9717264	ASP-3026-resistant ALK mutants	1/1	4.37 × 10^−5^	0.024	0.977
R-HSA-9717316	Alectinib-resistant ALK mutants	1/1	4.37 × 10^−5^	0.024	0.977
R-HSA-9717319	Brigatinib-resistant ALK mutants	1/1	4.37 × 10^−5^	0.024	0.977
R-HSA-9717326	Crizotinib-resistant ALK mutants	1/1	4.37 × 10^−5^	0.024	0.977
R-HSA-5657560	Hereditary fructose intolerance	1/2	8.73 × 10^−5^	0.048	0.977
R-HSA-3000157	Laminin interactions	3/34	0.001	0.052	0.977
R-HSA-5619085	Defective SLC26A3 causes DIAR1	1/3	1.31 × 10^−4^	0.071	0.977
R-HSA-5619076	Defective SLC17A8 causes DFNA25	1/3	1.31 × 10^−4^	0.071	0.977
R-HSA-5678420	Defective ABCC9 causes CMD10, ATFB12 and Cantu syndrome	1/3	1.31 × 10^−4^	0.071	0.977
R-HSA-5619045	Defective SLC34A2 causes PALM	1/3	1.31 × 10^−4^	0.071	0.977
R-HSA-9845622	Defective VWF binding to collagen type I	1/3	1.31 × 10^−4^	0.071	0.977
R-HSA-5687583	Defective SLC34A2 causes PALM	1/3	1.31 × 10^−4^	0.071	0.977

Abbreviations: ABCC9: ATP-binding cassette subfamily C member 9; ALK: anaplastic lymphoma kinase; ATFB12: atrial fibrillation familial 12; B3GALTL: beta 3-glucosyltransferase; CMD10: Dilated cardiomyopathy 10; DE genes: differentially expressed genes; DFNA25: autosomal dominant deafness 25; DIAR1: congenital secretory chloride diarrhea 1; FDR: false discovery rate; HSF1: heat shock transcription factor 1; PALM: pulmonary alveolar microlithiasis; PPs: Peters-Plus syndrome; SLC17A8: solute carrier family 17 member 8; SLC26A3: solute carrier family 26 member 3; SLC34A2: solute carrier family 34 member A2; TFAP2: transcription factor AP-2; TSR: thrombospondin type 1 repeat; VWF: Von Willebrand factor. * The *p*-value indicates the statistical significance of the DE genes associated with the Reactome pathways; while the FDR indicates the probability that the enrichment of these pathways is due to chance. For the complete analysis report of these pathways, see Supplementary File S3.

## Data Availability

All data are available in the text and in the Supplementary Files. The raw sequencing data have been deposited in the National Center for Biotechnology Information (NCBI) with the primary accession code “PRJNA1135143” and are available at: https://dataview.ncbi.nlm.nih.gov/object/PRJNA1135143?reviewer=nmempj6rem82dt8120mk1shn1u, accessed on 3 March 2025.

## Supplementary Materials

The following supporting information can be downloaded at: https://www.mdpi.com/article/10.3390/ijms26083662/s1. References [112,113,114,115,116,117,118,119,120,121,122,123,124,125,126,127,128,129,130,131,132,133,134,135,136,137,138,139,140,141,142,143,144,145,146,147,148,149,150,151,152,153,154,155,156,157,158,159,160,161] are mentioned in Supplementary File S3.

## Abbreviations

The following abbreviations are used in this manuscript:

*ATF3*	Activation of Transcription Factor 3
DBD	DNA binding domain
DISC	death-inducing signaling complex
DMEM	dulbecco’s Modified Eagle Medium
DMSO	dimethyl sulfoxide
DNA	deoxyribonucleic acid
EDTA	ethylene diamine tetraacetic acid
ER	estrogen receptor
FBS	fetal bovine serum
FDR	false discovery rate
GO	gene ontology
*HER-2*	human epidermal growth factor receptor 2
HSEs	heat shock elements
*HSF1*	Heat Shock Factor 1
HSP	heat shock protein
HSR	heat shock responsive
IC50	half maximal inhibitory concentration
VGKC	voltage-gated potassium channel
KLHL gene	Kelch-like gene
LZ	leucine zipper
PARP	poly ADP-ribose polymerase
PBS	phosphate-buffered saline
PD-1	programmed death-1
PD-L1	programmed death-ligand 1
PPI	protein–protein interaction
PR	progesterone receptor
RD	regulatory domain
RIN	RNA integrity number
RNA	ribonucleic acid
RNA-seq	RNA sequencing
RT-PCR	reverse transcription polymerase chain reaction
SLC	solute carrier
TAD	transactivation domain
TNBC	triple-negative breast cancer
VGCC	voltage-gated calcium channel
