# Supplementary material for: Identifying Genes Associated with the Anticancer Activity of a Fluorinated Chalcone in Triple-Negative Breast Cancer Cells Using Bioinformatics Tools
Source: Int J Mol Sci. 2025 Apr 12;26(8):3662. doi: 10.3390/ijms26083662 (PMC12027753; doi:10.3390/ijms26083662)
Supplement: Supplementary file 1 [file ijms-26-03662-s001.zip › ijms-3504982-supplementary.pdf]

reactome

Pathway Analysis Report

This report contains the pathway analysis results for the submitted sample ''. Analysis was performed against Reactome version 87 on 06/02/2024. The web link to these results is:

https://reactome.org/PathwayBrowser/#/ANALYSIS=MjAyNDAyMDYwNjM1MzZfMzM2NDE%3D

Please keep in mind that analysis results are temporarily stored on our server. The storage period depends on usage of the service but is at least 7 days. As a result, please note that this URL is only valid for a limited time period and it might have expired.

Table of Contents

1. Introduction
2. Properties
3. Genome-wide overview
4. Most significant pathways
5. Pathways details
6. Identifiers found
7. Identifiers not found

1. Introduction

Reactome is a curated database of pathways and reactions in human biology. Reactions can be considered as pathway 'steps'. Reactome defines a 'reaction' as any event in biology that changes the state of a biological molecule. Binding, activation, translocation, degradation and classical biochemical events involving a catalyst are all reactions. Information in the database is authored by expert biologists, entered and maintained by Reactome's team of curators and editorial staff. Reactome content frequently cross-references other resources e.g. NCBI, Ensembl, UniProt, KEGG (Gene and Compound), ChEBI, PubMed and GO. Orthologous reactions inferred from annotation for Homo sapiens are available for 14 non-human species including mouse, rat, chicken, puffer fish, worm, fly and yeast. Pathways are represented by simple diagrams following an SBGN-like format.

Reactome's annotated data describe reactions possible if all annotated proteins and small molecules were present and active simultaneously in a cell. By overlaying an experimental dataset on these annotations, a user can perform a pathway over-representation analysis. By overlaying quantitative expression data or time series, a user can visualize the extent of change in affected pathways and its progression. A binomial test is used to calculate the probability shown for each result, and the p-values are corrected for the multiple testing (Benjamini–Hochberg procedure) that arises from evaluating the submitted list of identifiers against every pathway.

To learn more about our Pathway Analysis, please have a look at our relevant publications:

Fabregat A, Sidiropoulos K, Garapati P, Gillespie M, Hausmann K, Haw R, … D'Eustachio P (2016). The reactome pathway knowledgebase. Nucleic Acids Research, 44(D1), D481–D487. https://doi.org/10.1093/nar/gkv1351.

Fabregat A, Sidiropoulos K, Viteri G, Forner O, Marin-Garcia P, Arnau V, … Hermjakob H (2017). Reactome pathway analysis: a high-performance in-memory approach. BMC Bioinformatics, 18.

2. Properties

- This is an **overrepresentation** analysis: A statistical (hypergeometric distribution) test that determines whether certain Reactome pathways are over-represented (enriched) in the submitted data. It answers the question ‘Does my list contain more proteins for pathway X than would be expected by chance?’ This test produces a probability score, which is corrected for false discovery rate using the Benjamani-Hochberg method.
- 189 out of 504 identifiers in the sample were found in Reactome, where 776 pathways were hit by at least one of them.
- All non-human identifiers have been converted to their human equivalent.
- IntAct interactors were included to increase the analysis background. This greatly increases the size of Reactome pathways, which maximises the chances of matching your submitted identifiers to the expanded pathway, but will include interactors that have not undergone manual curation by Reactome and may include interactors that have no biological significance, or unexplained relevance.
- This report is filtered to show only results for species 'Homo sapiens' and resource 'all resources'.
- The unique ID for this analysis (token) is MjAyNDAyMDYwNjM1MzZfMzM2NDE%3D. This ID is valid for at least 7 days in Reactome's server. Use it to access Reactome services with your data.

3. Genome-wide overview

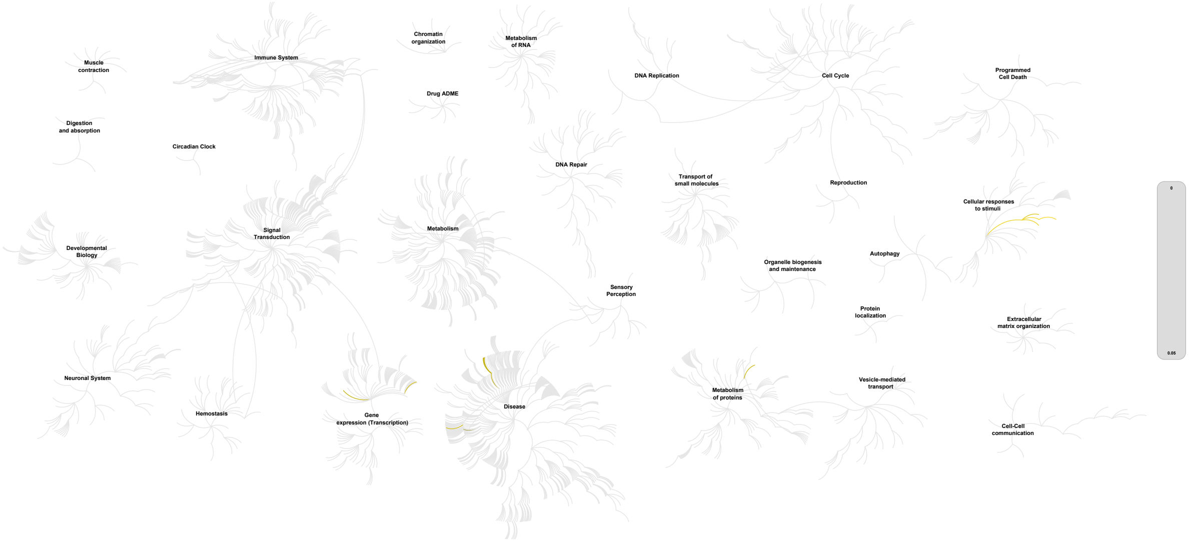

This figure shows a genome-wide overview of the results of your pathway analysis. Reactome pathways are arranged in a hierarchy. The center of each of the circular "bursts" is the root of one top-level pathway, for example "DNA Repair". Each step away from the center represents the next level lower in the pathway hierarchy. The color code denotes over-representation of that pathway in your input dataset. Light grey signifies pathways which are not significantly over-represented.

4. Most significant pathways

The following table shows the 25 most relevant pathways sorted by p-value.

Pathway name	Entities				Reactions	
	found	ratio	p-value	FDR*	found	ratio
HSF1-dependent transactivation	11 / 59	0.003	3.63e-07	2.96e-04	4 / 8	5.43e-04
Attenuation phase	9 / 47	0.002	3.37e-06	0.001	3 / 5	3.40e-04
Cellular response to heat stress	16 / 305	0.013	0.004	0.977	12 / 29	0.002
Regulation of HSF1-mediated heat shock response	14 / 260	0.011	0.006	0.977	7 / 14	9.51e-04
HSF1 activation	7 / 99	0.004	0.012	0.977	1 / 7	4.75e-04
Defective B3GALTL causes PpS	4 / 39	0.002	0.016	0.977	1 / 1	6.79e-05
TFAP2 (AP-2) family regulates transcription of other transcription factors	2 / 8	3.49e-04	0.017	0.977	2 / 2	1.36e-04
O-glycosylation of TSR domain-containing proteins	4 / 41	0.002	0.019	0.977	2 / 2	1.36e-04
Nuclear Receptor transcription pathway	7 / 113	0.005	0.023	0.977	2 / 2	1.36e-04
Drug resistance of ALK mutants	1 / 1	4.37e-05	0.024	0.977	7 / 7	4.75e-04
NVP-TAE684-resistant ALK mutants	1 / 1	4.37e-05	0.024	0.977	1 / 1	6.79e-05
ceritinib-resistant ALK mutants	1 / 1	4.37e-05	0.024	0.977	1 / 1	6.79e-05
lorlatinib-resistant ALK mutants	1 / 1	4.37e-05	0.024	0.977	1 / 1	6.79e-05
ASP-3026-resistant ALK mutants	1 / 1	4.37e-05	0.024	0.977	1 / 1	6.79e-05
alectinib-resistant ALK mutants	1 / 1	4.37e-05	0.024	0.977	1 / 1	6.79e-05
brigatinib-resistant ALK mutants	1 / 1	4.37e-05	0.024	0.977	1 / 1	6.79e-05
crizotinib-resistant ALK mutants	1 / 1	4.37e-05	0.024	0.977	1 / 1	6.79e-05
Hereditary fructose intolerance	1 / 2	8.73e-05	0.048	0.977	1 / 1	6.79e-05
Laminin interactions	3 / 34	0.001	0.052	0.977	12 / 15	0.001
Defective SLC26A3 causes congenital secretory chloride diarrhea 1 (DIAR1)	1 / 3	1.31e-04	0.071	0.977	1 / 1	6.79e-05
Defective SLC17A8 causes autosomal dominant deafness 25 (DFNA25)	1 / 3	1.31e-04	0.071	0.977	1 / 1	6.79e-05
Defective ABCC9 causes CMD10, ATFB12 and Cantu syndrome	1 / 3	1.31e-04	0.071	0.977	1 / 1	6.79e-05
Defective SLC34A2 causes pulmonary alveolar microlithiasis (PALM)	1 / 3	1.31e-04	0.071	0.977	1 / 1	6.79e-05
Defective VWF binding to collagen type I	1 / 3	1.31e-04	0.071	0.977	1 / 1	6.79e-05
Defective SLC34A2 causes PALM	1 / 3	1.31e-04	0.071	0.977	1 / 1	6.79e-05

* False Discovery Rate

5. Pathways details

For every pathway of the most significant pathways, we present its diagram, as well as a short summary, its bibliography and the list of inputs found in it.

1. HSF1-dependent transactivation (R-HSA-3371571)

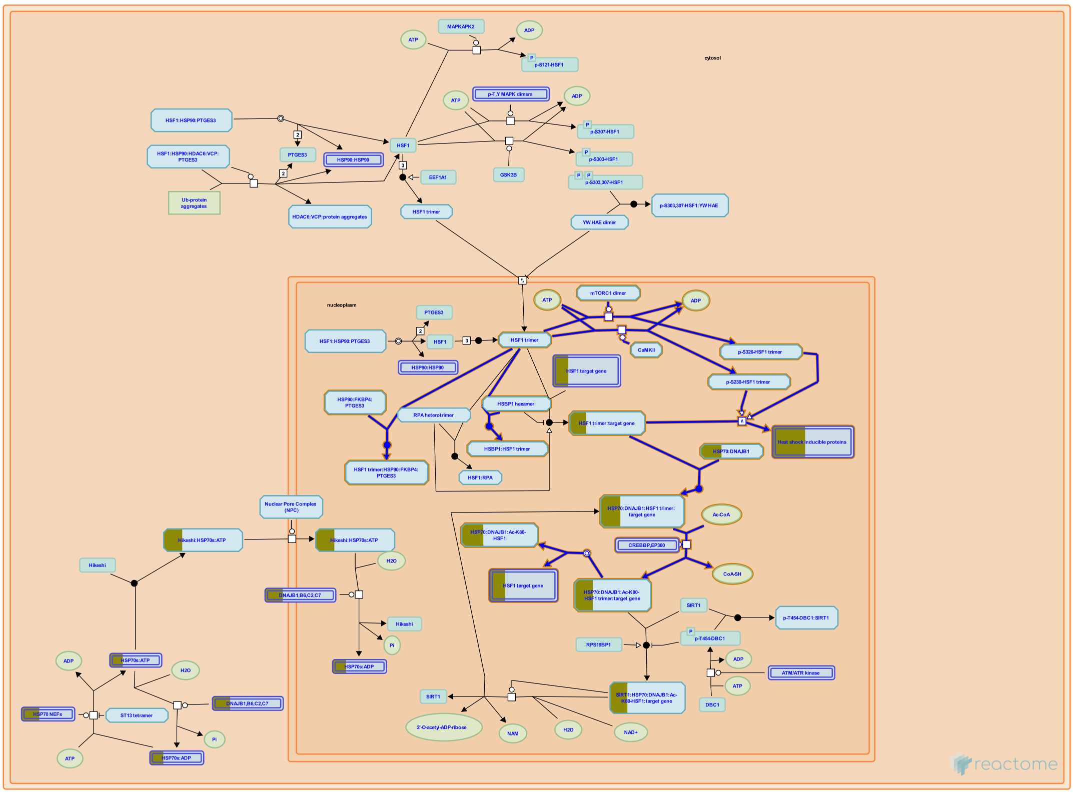

Acquisition of DNA binding activity by HSF1 is necessary but insufficient for transcriptional activation (Cotto JJ et al. 1996; Trinklein ND et al. 2004). In addition to having a sequence-specific DNA binding domain, HSF1 contains a C-terminal region which is involved in activating the transcription of the target genes (Green M et al. 1995). However, the transactivating ability of the transactivation domain itself is not stress sensitive. Rather, it's controled by a regulatory domain of HSF1 (amino acids 221-310), which represses the transactivating ability under normal physiological conditions (Green M et al. 1995; Zuo J et al. 1995; Newton EM et al. 1996). The HSF1 transactivation domain can be divided into two distinct regions, activation domain 1 (AD1) and activation domain 2 (AD2) (Brown SA et al. 1998). AD1 and AD2 each contain residues that are important for both transcriptional initiation and elongation. Mutations in acidic residues in both AD1 and AD2 preferentially affect the ability of HSF1 to stimulate transcriptional initiation, while mutations in phenylalanine residues preferentially affect stimulation of elongation (Brown SA et al. 1998).

Activation of the DNA-bound but transcriptionally incompetent HSF1 is thought to occur upon stress induced HSF1 phosphorylation at several serine residues (Ding XZ et al. 1997; Holmberg CI et al. 2001; Guettouche T et al. 2005). In cells exposed to heat, acquisition of HSE DNA-binding activity was observed to precede phosphorylation of HSF1 (Cotto JJ et al. 1996; Kline MP & Morimoto RI 1997). While there is a sufficient evidence to suggest that phosphorylation of HSF1 is essential to modulate HSF1 transactiviting capacity, mechanisms behind stress stimuli and kinases/phosphatases involved have not been clearly established.

References

Rungger D, Voellmy R & Zuo J (1995). Multiple layers of regulation of human heat shock transcription factor 1. Mol. Cell. Biol., 15, 4319-30.

Edit history

Date	Action	Author
2013-05-13	Created	Shamovsky V
2013-10-29	Authored	Shamovsky V
2014-02-17	Edited	Shamovsky V
2014-02-17	Reviewed	Pani B
2023-11-17	Modified	Wright A

9 submitted entities found in this pathway, mapping to 11 Reactome entities

Input	UniProt Id	Input	UniProt Id
ENSG00000109846	P02511	ENSG00000132002	P25685
ENSG00000152137	Q9UJY1	ENSG00000204389	P0DMV8

Input	Ensembl Id	Input	Ensembl Id	Input	Ensembl Id
ENSG00000106211	ENSG00000106211	ENSG00000120694	ENSG00000120694	ENSG00000132002	ENSG00000132002
ENSG00000149257	ENSG00000149257	ENSG00000160570	ENSG00000160570	ENSG00000173110	ENSG00000173110
ENSG00000204389	ENSG00000204389				

2. Attenuation phase (R-HSA-3371568)

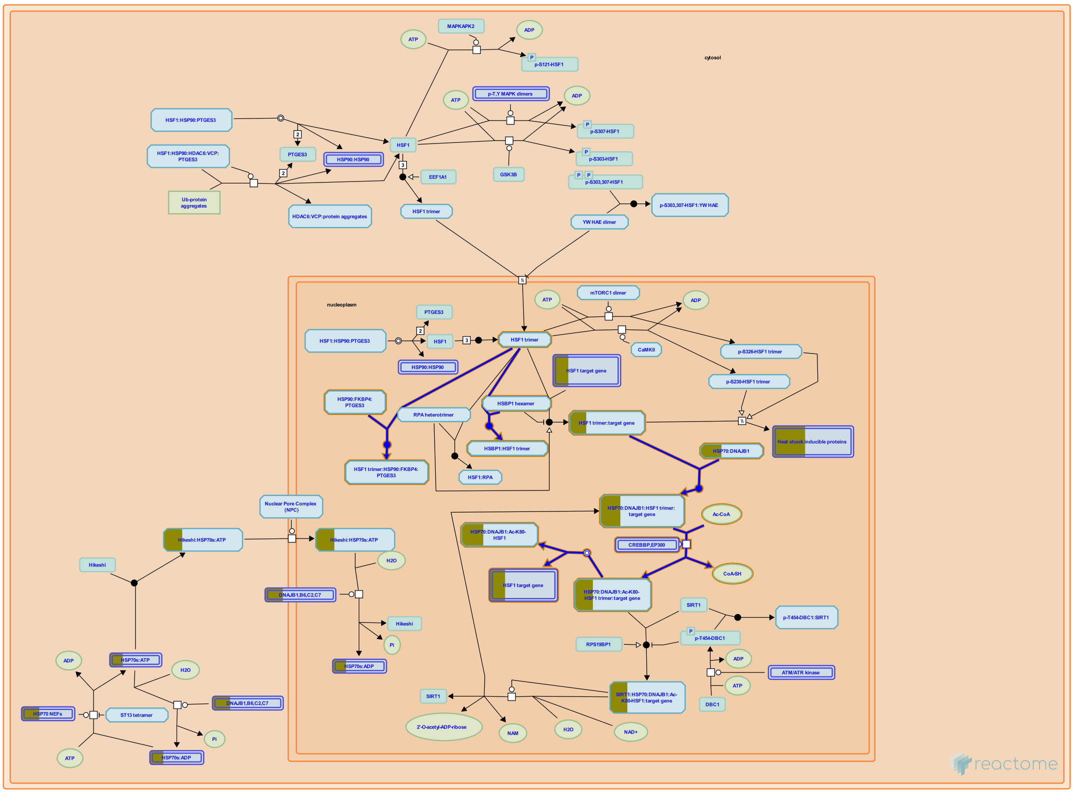

Attenuation of the heat shock transcriptional response occurs during continuous exposure to intermediate heat shock conditions or upon recovery from stress (Abravaya et al. 1991). The attenuation phase of HSF1 cycle involves the transcriptional silencing of HSF1 bound to HSE, the release of HSF1 trimers from HSE and dissociation of HSF1 trimers to monomers. HSF1-driven heat stress associated transcription was shown to depend on inducible and reversible acetylation of HSF1 at Lys80, which negatively regulates DNA binding activity of HSF1 (Westerheide SD et al. 2009). In addition, the attenuation of HSF1 activation takes place when enough HSP70/HSP40 is produced to saturate exposed hydrophobic regions of proteins damaged as a result of heat exposure. The excess HSP70/HSP40 binds to HSF1 trimer, which leads to its dissociation from the promoter and conversion to the inactive monomeric form (Abravaya et al. 1991; Shi Y et al. 1998). Interaction of HSP70 with the transcriptional corepressor repressor element 1-silencing transcription factor corepressor (CoREST) assists in terminating heat-shock response (Gomez AV et al. 2008). HSF1 DNA-binding and transactivation activity were also inhibited upon interaction of HSF1-binding protein (HSBP1) with active trimeric HSF1(Satyal SH et al. 1998).

References

Morimoto RI, Abravaya K & Phillips B (1991). Attenuation of the heat shock response in HeLa cells is mediated by the release of bound heat shock transcription factor and is modulated by changes in growth and in heat shock temperatures. Genes Dev., 5, 2117-27.

Edit history

Date	Action	Author
2013-05-13	Created	Shamovsky V
2013-10-29	Authored	Shamovsky V

Date	Action	Author
2014-02-17	Edited	Shamovsky V
2014-02-17	Reviewed	Pani B
2023-11-16	Modified	Wright A

7 submitted entities found in this pathway, mapping to 9 Reactome entities

Input	UniProt Id	Input	UniProt Id
ENSG00000132002	P25685	ENSG00000204389	P0DMV8

Input	Ensembl Id	Input	Ensembl Id	Input	Ensembl Id
ENSG00000106211	ENSG00000106211	ENSG00000120694	ENSG00000120694	ENSG00000132002	ENSG00000132002
ENSG00000149257	ENSG00000149257	ENSG00000160570	ENSG00000160570	ENSG00000173110	ENSG00000173110
ENSG00000204389	ENSG00000204389				

3. Cellular response to heat stress (R-HSA-3371556)

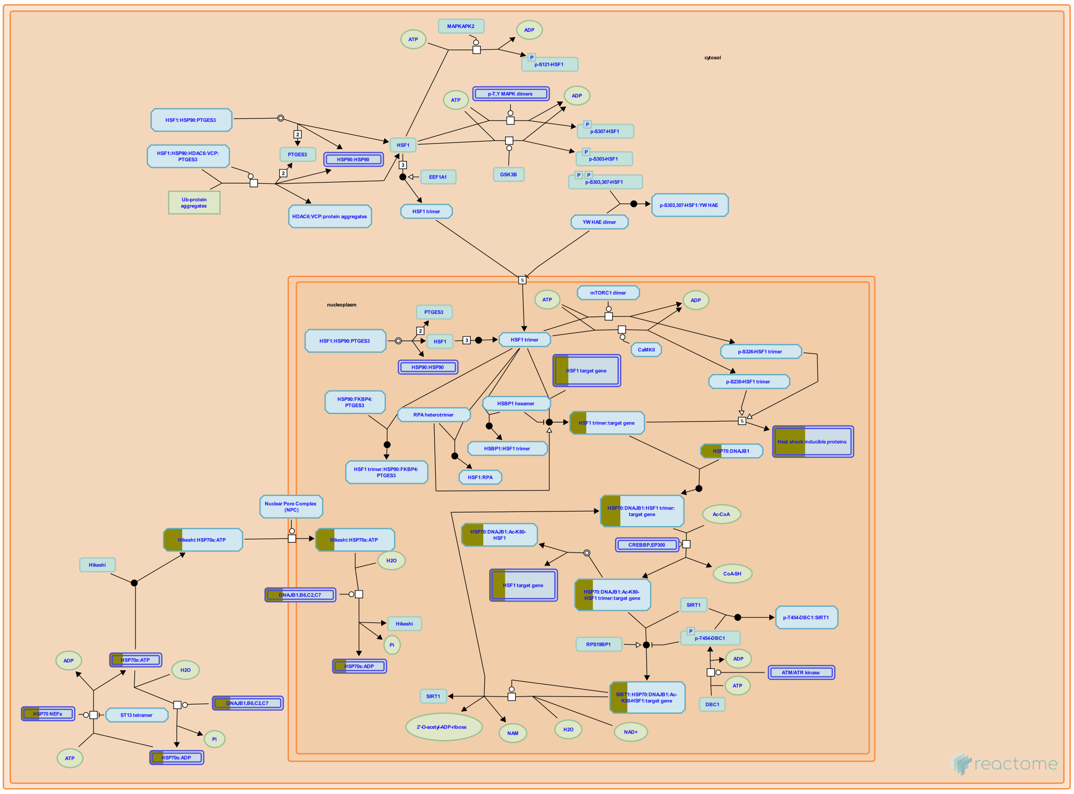

In response to exposure to elevated temperature and certain other proteotoxic stimuli (e.g., hypoxia, free radicals) cells activate a number of cytoprotective mechanisms known collectively as "heat shock response". Major aspects of the heat shock response (HSR) are evolutionarily conserved events that allow cells to recover from protein damage induced by stress (Liu XD et al. 1997; Voellmy R & Boellmann F 2007; Shamovsky I & Nudler E 2008; Anckar J & Sistonen L 2011). The main hallmark of HSR is the dramatic alteration of the gene expression pattern. A diverse group of protein genes is induced by the exposure to temperatures 3-5 degrees higher than physiological. Functionally, most of these genes are molecular chaperones that ensure proper protein folding and quality control to maintain cell proteostasis.

At the same time, heat shock-induced phosphorylation of translation initiation factor eIF2alpha leads to the shutdown of the nascent polypeptide synthesis reducing the burden on the chaperone system that has to deal with the increased amount of misfolded and thermally denatured proteins (Duncan RF & Hershey JWB 1989; Sarkar A et al. 2002; Spriggs KA et al. 2010).

The induction of HS gene expression primarily occurs at the level of transcription and is mediated by heat shock transcription factor HSF1(Sarge KD et al. 1993; Baler R et al. 1993). Human cells express five members of HSF protein family: HSF1, HSF2, HSF4, HSFX and HSFY. HSF1 is the master regulator of the heat inducible gene expression (Zuo J et al. 1995; Akerfelt M et al. 2010). HSF2 is activated in response to certain developmental stimuli in addition to being co-activated with HSF1 to provide promoter-specific fine-tuning of the HS response by forming heterotrimers with HSF1 (Ostling P et al. 2007; Sandqvist A et al. 2009). HSF4 lacks the transcription activation domain and acts as a repressor of certain genes during HS (Nakai A et al. 1997; Tanabe M et al. 1999; Kim SA et al. 2012). Two additional family members HSFX and HSFY, which are located on the X and Y chromosomes respectively, remain to be characterized (Bhowmick BK et al. 2006; Shinka T et al. 2004; Kichine E et al. 2012).

Under normal conditions HSF1 is present in both cytoplasm and nucleus in the form of an inactive monomer. The monomeric state of HSF1 is maintained by an intricate network of protein-protein interactions that include the association with HSP90 multichaperone complex, HSP70/HSP40 chaperone machinery, as well as intramolecular interaction of two conserved hydrophobic repeat regions. Monomeric HSF1 is constitutively phosphorylated on Ser303 and Ser 307 by (Zou J et al. 1998; Knauf U et al. 1996; Kline MP & Moromoto RI 1997; Guettouche T et al. 2005). This phosphorylation plays an essential role in ensuring cytoplasmic localization of at least a subpopulation of HSF1 molecules under normal conditions (Wang X et al. 2004).

Exposure to heat and other proteotoxic stimuli results in the release of HSF1 from the inhibitory complex with chaperones and its subsequent trimerization, which is promoted by its interaction with translation elongation factor eEF1A1 (Baler R et al. 1993; Shamovsky I et al. 2006; Herbomel G et al 2013). The trimerization is believed to involve intermolecular interaction between hydrophobic repeats 1-3 leading to the formation of a triple coil structure. Additional stabilization of the HSF1 trimer is provided by the formation of intermolecular S-S bonds between Cys residues in the DNA binding domain (Lu M et al.2008). Trimeric HSF1 is predominantly localized in the nucleus where it binds the specific sequence in the promoter of hsp genes (Sarge KD et al. 1993; Wang Y and Morgan WD 1994). The binding sequence for HSF1 (HSE, heat shock element) contains series of inverted repeats nGAAn in head-to-tail orientation, with at least three elements being required for the high affinity binding. Binding of the HSF1 trimer to the promoter is not sufficient to induce transcription of the gene (Cotto J et al. 1996). In order to do so, HSF1 needs to undergo inducible phosphorylation on specific Ser residues such as Ser230, Ser326. This phosphorylated form of HSF1 trimer is capable of increasing the promoter initiation rate. HSF1 bound to DNA promotes recruiting components of the transcription mediator complex and relieving promoter-proximal pause of RNA polymerase II through its interaction with TFIIH transcription factor (Yuan CX & Gurley WB 2000).

HSF1 activation is regulated in a precise and tight manner at multiple levels (Zuo J et al. 1995; Cotto J et al. 1996). This allows fast and robust activation of HS response to minimize proteotoxic effects of the stress. The exact set of HSF1 inducible genes is probably cell type specific. Moreover, cells in different pathophysiological states will display different but overlapping profile of HS inducible genes.

References

Voellmy R, Baler R & Dahl G (1993). Activation of human heat shock genes is accompanied by oligomerization, modification, and rapid translocation of heat shock transcription factor HSF1. Mol. Cell. Biol., 13, 2486-96.

Murphy SP, Morimoto RI & Sarge KD (1993). Activation of heat shock gene transcription by heat shock factor 1 involves oligomerization, acquisition of DNA-binding activity, and nuclear localization and can occur in the absence of stress. Mol. Cell. Biol., 13, 1392-407.

Edit history

Date	Action	Author
2013-05-13	Created	Shamovsky V
2013-10-29	Authored	Shamovsky V
2014-02-17	Edited	Shamovsky V
2014-02-17	Reviewed	Pani B

Date	Action	Author
2023-11-17	Modified	Wright A

12 submitted entities found in this pathway, mapping to 16 Reactome entities

Input	UniProt Id	Input	UniProt Id	Input	UniProt Id
ENSG00000109846	P02511	ENSG00000120694	Q92598	ENSG00000132002	P25685
ENSG00000151929	O95817	ENSG00000152137	Q9UJY1	ENSG00000164070	O95757
ENSG00000173110	P17066	ENSG00000204389	P0DMV8	ENSG00000225217	P48741

Input	Ensembl Id	Input	Ensembl Id	Input	Ensembl Id
ENSG00000106211	ENSG00000106211	ENSG00000120694	ENSG00000120694	ENSG00000132002	ENSG00000132002
ENSG00000149257	ENSG00000149257	ENSG00000160570	ENSG00000160570	ENSG00000173110	ENSG00000173110
ENSG00000204389	ENSG00000204389				

4. Regulation of HSF1-mediated heat shock response (R-HSA-3371453)

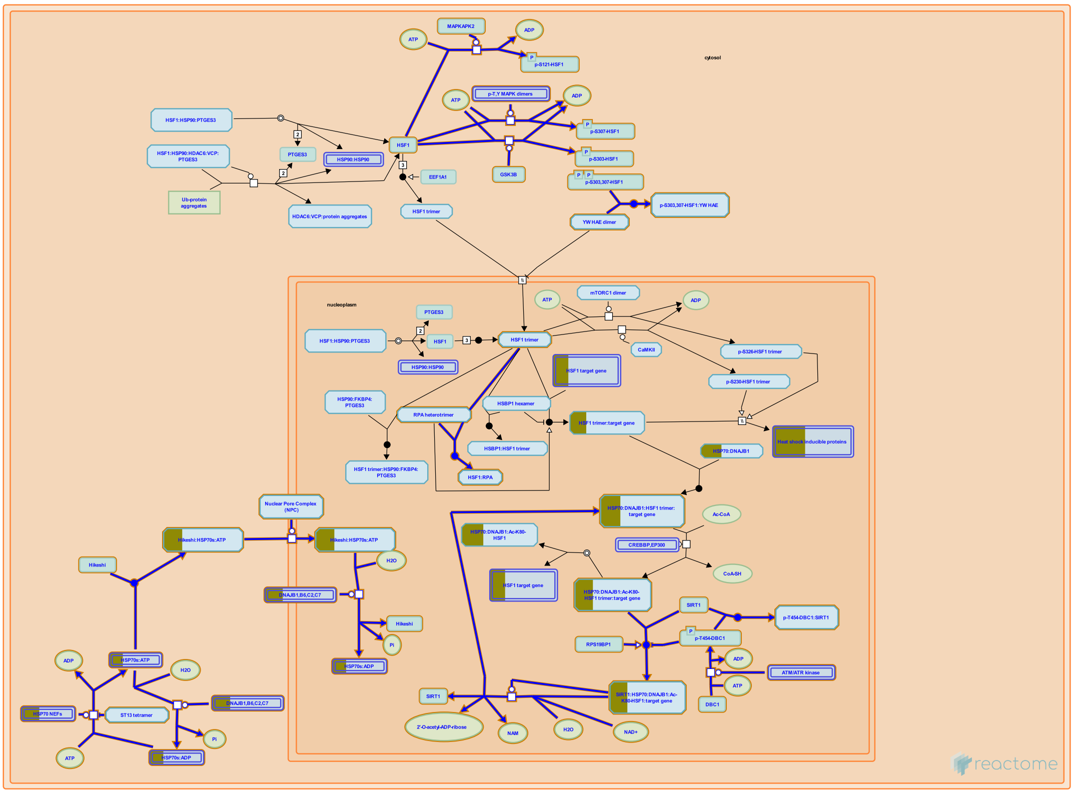

The ability of HSF1 to respond to cellular stresses is under negative regulation by chaperones, modulation of nucleocytoplasmic shuttling, post-translational modifications and transition from monomeric to trimeric state.

References

Rungger D, Voellmy R & Zuo J (1995). Multiple layers of regulation of human heat shock transcription factor 1. Mol. Cell. Biol., 15, 4319-30.

Edit history

Date	Action	Author
2013-05-13	Created	Shamovsky V
2013-10-29	Authored	Shamovsky V
2014-02-17	Edited	Shamovsky V
2014-02-17	Reviewed	Pani B
2023-11-17	Modified	Wright A

10 submitted entities found in this pathway, mapping to 14 Reactome entities

Input	UniProt Id	Input	UniProt Id	Input	UniProt Id
ENSG00000120694	Q92598	ENSG00000132002	P25685	ENSG00000151929	O95817
ENSG00000164070	O95757	ENSG00000173110	P17066	ENSG00000204389	P0DMV8
ENSG00000225217	P48741				

Input	Ensembl Id	Input	Ensembl Id	Input	Ensembl Id
ENSG00000106211	ENSG00000106211	ENSG00000120694	ENSG00000120694	ENSG00000132002	ENSG00000132002
ENSG00000149257	ENSG00000149257	ENSG00000160570	ENSG00000160570	ENSG00000173110	ENSG00000173110
ENSG00000204389	ENSG00000204389				

5. HSF1 activation (R-HSA-3371511)

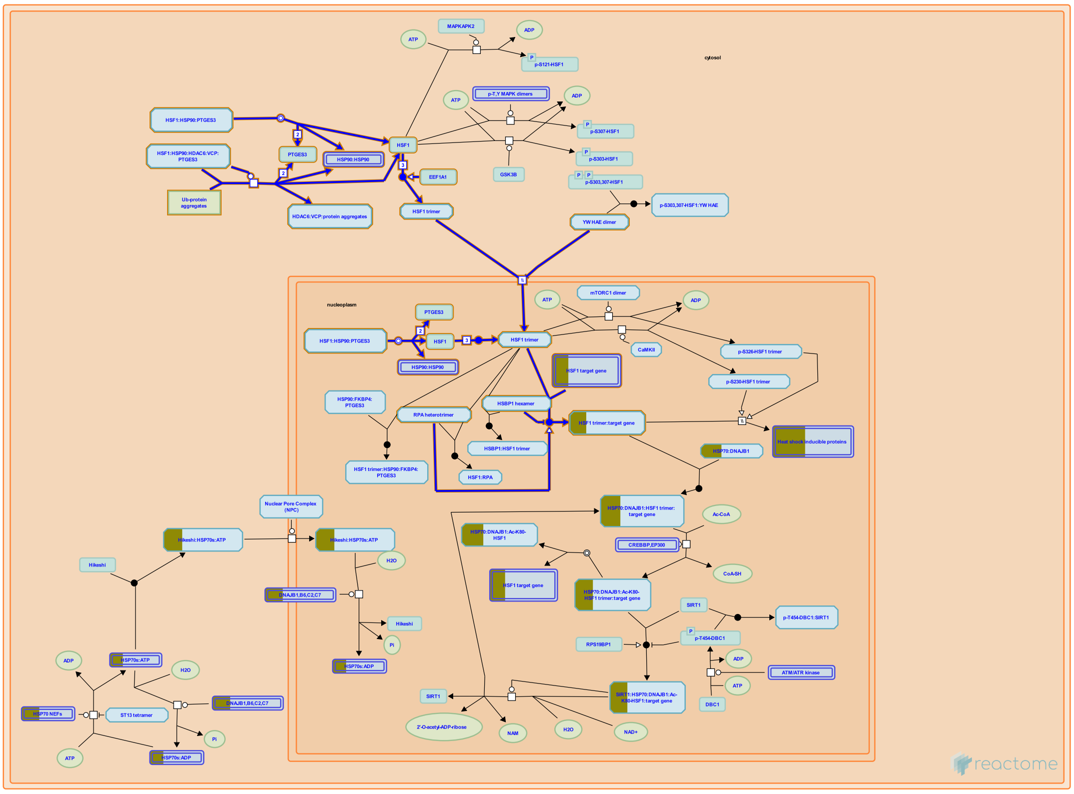

Heat shock factor 1 (HSF1) is a transcription factor that activates gene expression in response to a variety of stresses, including heat shock, oxidative stress, as well as inflammation and infection (Shamovsky I and Nudler E 2008; Akerfelt et al. 2010; Bjork and Sistonen 2010; Anckar and Sistonen 2011).

HSF1 is constitutively present in the cell. In the absence of stress HSF1 is found in both the cytoplasm and the nucleus as an inactive monomer (Sarge KD et al. 1993; Mercier PA et al. 1999; Vujanac M et al. 2005). A physical or chemical proteotoxic stress rapidly induces HSF1 activation, which occurs through a multi?step process, involving HSF1 monomer-to-homotrimer transition, nuclear accumulation, and binding to a promoter element, called the heat shock element (HSE), which leads to the increase in the stress-inducible gene expression (Sarge KD et al. 1993; Baler R et al. 1998; Sonna LA et al. 2002; Shamovsky I and Nudler E 2008; Sakurai H and Enoki Y 2010; Herbomel G et al. 2013). Depending on the type of stress stimulus, the multiple events associated with HSF1 activation might be affected differently (Holmberg CI et al 2000; Bjork and Sistonen 2010).

References

Morimoto RI, Kline M & Cotto JJ (1996). Activation of heat shock factor 1 DNA binding precedes stress-induced serine phosphorylation. Evidence for a multistep pathway of regulation. J. Biol. Chem., 271, 3355-8.

Rungger D, Voellmy R & Zuo J (1995). Multiple layers of regulation of human heat shock transcription factor 1. Mol. Cell. Biol., 15, 4319-30.

Edit history

Date	Action	Author
2013-05-13	Created	Shamovsky V
2013-10-29	Authored	Shamovsky V
2014-02-17	Edited	Shamovsky V
2014-02-17	Reviewed	Pani B
2023-11-17	Modified	Wright A

7 submitted entities found in this pathway, mapping to 7 Reactome entities

Input	Ensembl Id	Input	Ensembl Id	Input	Ensembl Id
ENSG00000106211	ENSG00000106211	ENSG00000120694	ENSG00000120694	ENSG00000132002	ENSG00000132002
ENSG00000149257	ENSG00000149257	ENSG00000160570	ENSG00000160570	ENSG00000173110	ENSG00000173110
ENSG00000204389	ENSG00000204389				

6. Defective B3GALTL causes PpS (R-HSA-5083635)

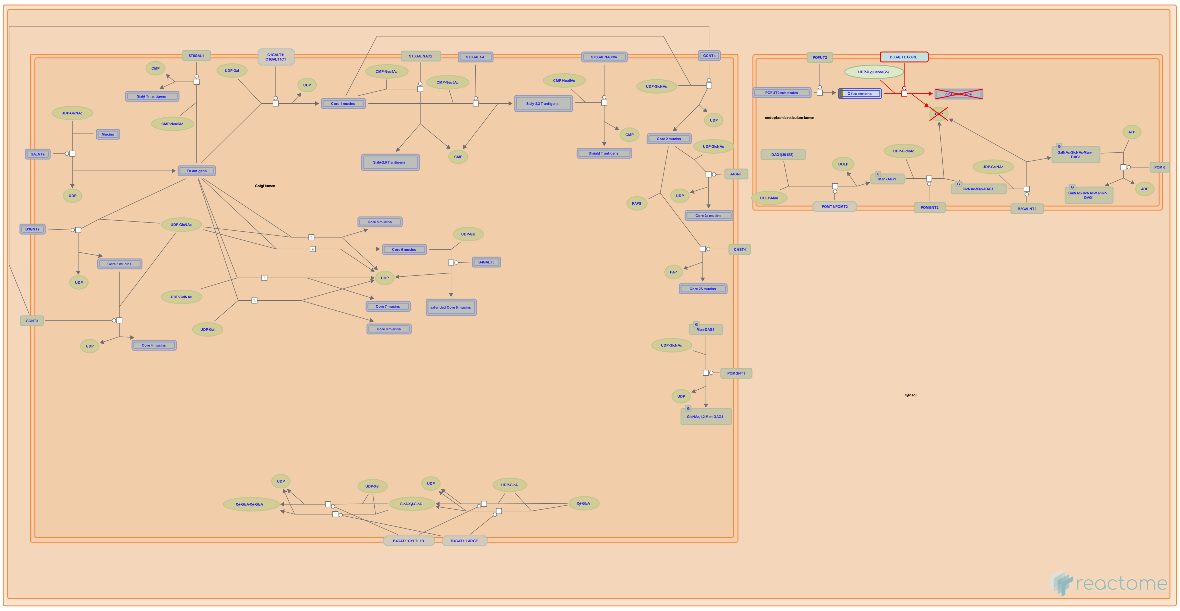

Diseases: eye disease, orofacial cleft.

Human beta-1,3-glucosyltransferase like protein (B3GALTL, HGNC Approved Gene Symbol: B3GLCT; MIM:610308; CAZy family GT31), localised on the ER membrane, glucosylates O-fucosylated proteins. The resultant glc-beta-1,3-fuc disaccharide modification on thrombospondin type 1 repeat (TSR1) domain-containing proteins is thought to assist in the secretion of many of these proteins from the ER lumen, and mediate an ER quality-control mechanism of folded TSRs (Vasudevan et al. 2015). Defects in B3GALTL can cause Peters plus syndrome (PpS; MIM:261540), an autosomal recessive disorder characterised by anterior eye chamber defects, short stature, delay in growth and mental developmental and cleft lip and/or palate (Heinonen & Maki 2009).

References

Vasudevan D, Haltiwanger RS, Johar SS, Takeuchi H & Majerus E (2015). Peters plus syndrome mutations disrupt a noncanonical ER quality-control mechanism. Curr. Biol., 25, 286-95.

Maki M & Heinonen TY (2009). Peters'-plus syndrome is a congenital disorder of glycosylation caused by a defect in the beta1,3-glucosyltransferase that modifies thrombospondin type 1 repeats. Ann. Med., 41, 2-10.

Edit history

Date	Action	Author
2013-11-07	Edited	Jassal B
2013-11-07	Authored	Jassal B
2013-11-07	Created	Jassal B
2015-12-18	Reviewed	Hansen L, Joshi HJ
2023-10-12	Modified	Weiser JD

4 submitted entities found in this pathway, mapping to 4 Reactome entities

Input	UniProt Id	Input	UniProt Id
ENSG00000140873	Q8TE60	ENSG00000154734	Q9UHI8
ENSG00000173157	P59510	ENSG00000262655	Q9HCB6

7. TFAP2 (AP-2) family regulates transcription of other transcription factors (R-HSA-8866906)

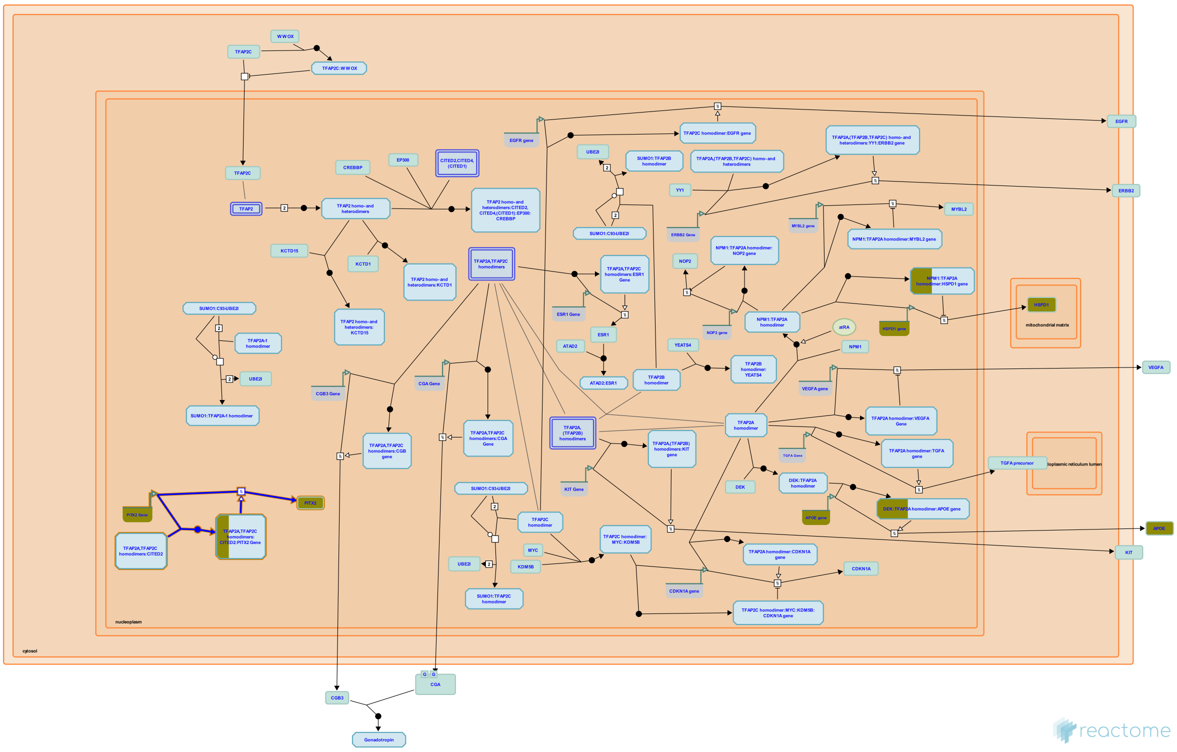

Homodimers and possibly heterodimers of TFAP2A and TFAP2C, in complex with CITED2, stimulate transcription of the PITX2 gene, involved in left-right patterning and heart development (Bamforth et al. 2004, Li et al. 2012).

References

Anderson RH, Bhattacharya S, Farthing CR, Broadbent C, Neubauer S, Bragança J, ... Brown NA (2004). Cited2 controls left-right patterning and heart development through a Nodal-Pitx2c pathway. Nat. Genet., 36, 1189-96.

Pan H, Guan L, Li Q, Su D & Ma X (2012). CITED2 mutation links congenital heart defects to dysregulation of the cardiac gene VEGF and PITX2C expression. Biochem. Biophys. Res. Commun., 423, 895-9.

Edit history

Date	Action	Author
2016-03-14	Edited	Orlic-Milacic M
2016-03-14	Authored	Orlic-Milacic M
2016-04-04	Created	Orlic-Milacic M
2016-05-04	Reviewed	Dawid IB, Zarelli VE
2016-05-17	Reviewed	Bogachek MV, Weigel RJ
2023-03-08	Modified	Matthews L

1 submitted entities found in this pathway, mapping to 2 Reactome entities

Input	UniProt Id
ENSG00000164093	Q99697

Input	Ensembl Id
ENSG00000164093	ENSG00000164093

8. O-glycosylation of TSR domain-containing proteins (R-HSA-5173214)

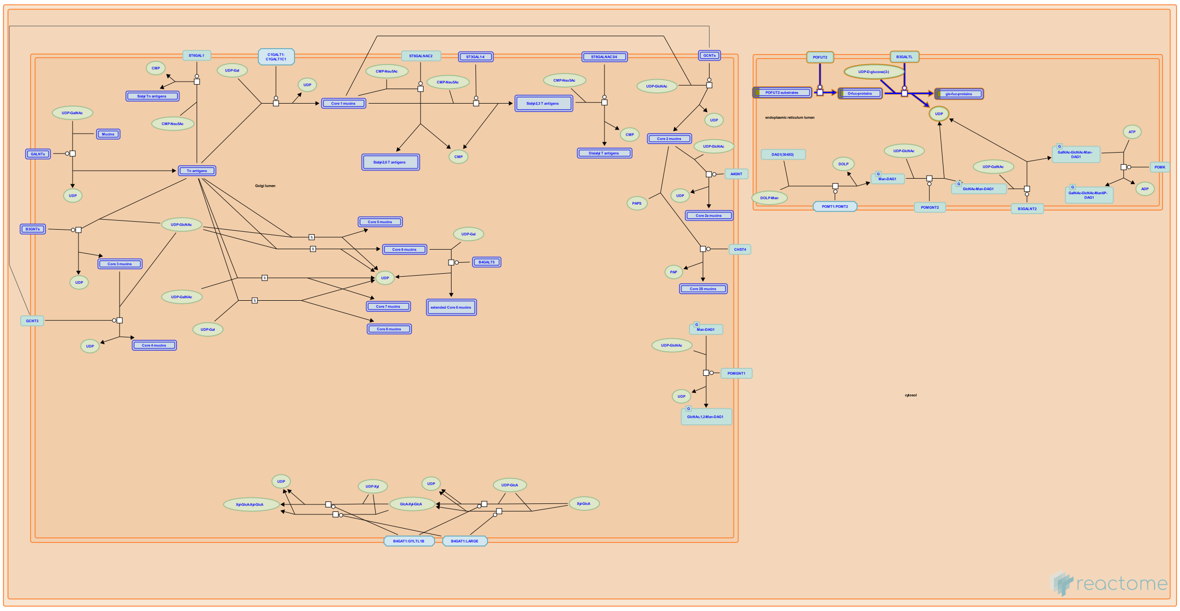

The O-fucosylation of proteins containing thrombospondin type 1 repeat (TSR) domains is an important PTM, regulating many biological processes such as Notch signalling, inflammation, wound healing, angiogenesis amd neoplasia (Adams & Tucker 2000, Moremen et al. 2012). Fucose addition is carried out by two protein fucosyltransferases, POFUT1 and 2. Only POFUT2 recognises the consensus sequence CSXS/TCG found in TSR1 domains and the fucosyl residue is attached to the hydroxyl group of conserved serine (S) or threonine (T) residues within the consensus sequence. The modification was first demonstrated on thrombospondin 1, found in platelets and the ECM (Hofsteenge et al. 2001, Luo et al. 2006). The resulting O-fucosyl-protein is subsequently a substrate for beta-1,3-glucosyltransferase-like protein (B3GALTL), which adds a glucosyl moiety to form the rare disaccharide modification Glc-beta-1,3-Fuc. More than 60 human proteins contain TSR1 domains, The disaccharide modification has been demonstrated on a small number of these TSR1 domain-containing proteins such as thrombospondin 1 (Hofsteenge et al. 2001, Luo et al. 2006), properdin (Gonzalez de Peredo et al. 2002) and F-spondin (Gonzalez de Peredo et al. 2002). The ADAMTS (a disintegrin-like and metalloprotease domain with thrombospondin type-1 repeats) superfamily consists of 19 secreted metalloproteases (ADAMTS proteases) and at lease five ADAMTS-like proteins in humans. Five members of the ADAMTS superfamily have also had experimental confirmation of the disaccharide modification. Examples are ADAMTS13 (Ricketts et al. 2007) and ADAMTSL1 (Wang et al. 2007). In the two reactions described here, the TSR1 domain-containing proteins with similarity to the experimentally confirmed ones are included as putative substrates.

References

Haltiwanger RS, Dlugosz M, Luther KB, Majerus EM & Ricketts LM (2007). O-fucosylation is required for ADAMTS13 secretion. J. Biol. Chem., 282, 17014-23.

Adams JC & Tucker RP (2000). The thrombospondin type 1 repeat (TSR) superfamily: diverse proteins with related roles in neuronal development. Dev. Dyn., 218, 280-99.

Haltiwanger RS, Nita-Lazar A & Luo Y (2006). Two distinct pathways for O-fucosylation of epidermal growth factor-like or thrombospondin type 1 repeats. J. Biol. Chem., 281, 9385-92.

Huwiler KG, Mosher DF, Hofsteenge J, Lawler J, Hess D, Macek B & Peter-Katalinic J (2001). C-mannosylation and O-fucosylation of the thrombospondin type 1 module. J. Biol. Chem., 276, 6485-98.

Hofsteenge J, Hess D, Klein D, Gonzalez de Peredo A, Peter-Katalinic J & Macek B (2002). C-mannosylation and o-fucosylation of thrombospondin type 1 repeats. Mol. Cell Proteomics, 1, 11-8.

Edit history

Date	Action	Author
2013-11-25	Edited	Jassal B
2013-11-25	Authored	Jassal B
2013-11-25	Created	Jassal B
2014-02-07	Reviewed	D'Eustachio P
2023-11-17	Modified	Wright A

4 submitted entities found in this pathway, mapping to 4 Reactome entities

Input	UniProt Id	Input	UniProt Id
ENSG00000140873	Q8TE60	ENSG00000154734	Q9UHI8
ENSG00000173157	P59510	ENSG00000262655	Q9HCB6

9. Nuclear Receptor transcription pathway (R-HSA-383280)

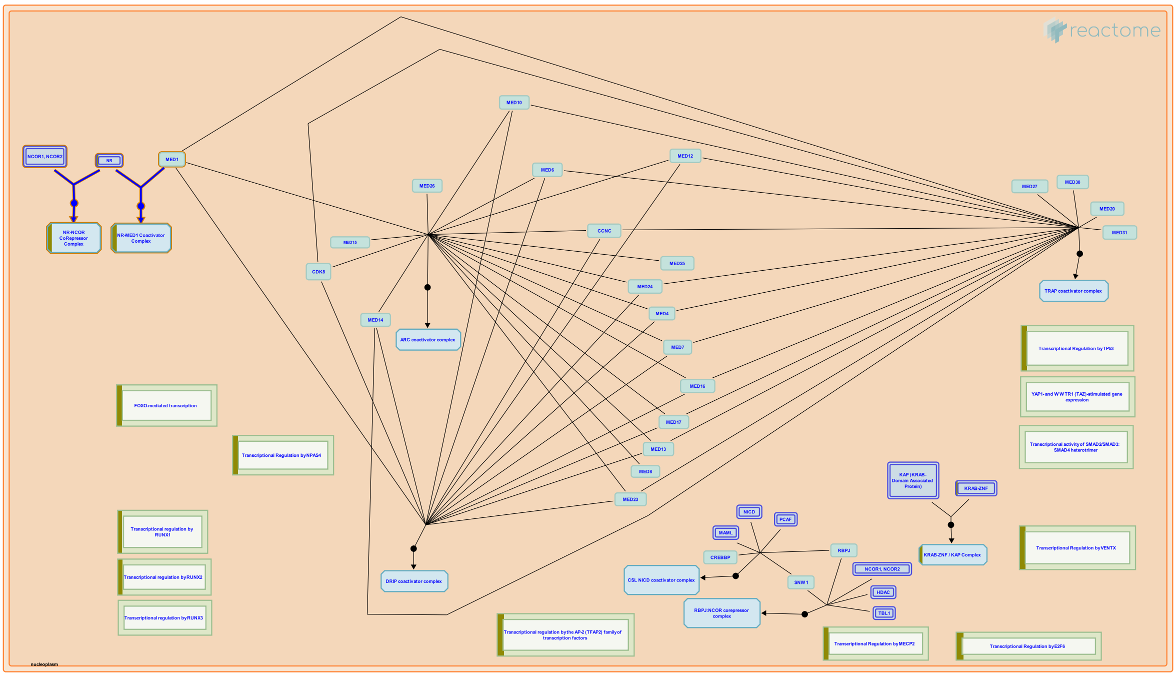

A classic example of bifunctional transcription factors is the family of Nuclear Receptor (NR) proteins. These are DNA-binding transcription factors that bind certain hormones, vitamins, and other small, diffusible signaling molecules. The non-liganded NRs recruit specific corepressor complexes of the NCOR/SMRT type, to mediate transcriptional repression of the target genes to which they are bound. During signaling, ligand binding to a specific domain the NR proteins induces a conformational change that results in the exchange of the associated CoR complex, and its replacement by a specific coactivator complex of the TRAP / DRIP / Mediator type. These coactivator complexes typically nucleate around a MED1 coactivator protein that is directly bound to the NR transcription factor.

A general feature of the 49 human NR proteins is that in the unliganded state, they each bind directly to an NCOR corepressor protein, either NCOR1 or NCOR2 (NCOR2 was previously named "SMRT"). This NCOR protein nucleates the assembly of additional, specific corepressor proteins, depending on the cell and DNA context. The NR-NCOR interaction is mediated by a specific protein interaction domain (PID) present in the NRs that binds to specific cognate PID(s) present in the NCOR proteins. Thus, the human NRs each take part in an NR-NCOR binding reaction in the absence of binding by their ligand.

A second general feature of the NR proteins is that they each contain an additional, but different PID that mediates specific binding interactions with MED1 proteins. In the ligand-bound state, NRs each take part in an NR-MED1 binding reaction to form an NR-MED1 complex. The bound MED1 then functions to nucleate the assembly of additional specific coactivator proteins, depending on the cell and DNA context, such as what specific target gene promoter they are bound to, and in what cell type.

The formation of specific MED1-containing coactivator complexes on specific NR proteins has been well-characterized for a number of the human NR proteins (see Table 1 in (Bourbon, 2004)). For example, binding of thyroid hormone (TH) to the human TH Receptor (THRA or THRB) was found to result in the recruitment of a specific complex of Thyroid Receptor Associated Proteins - the TRAP coactivator complex - of which the TRAP220 subunit was later identified to be the Mediator 1 (MED1) homologue.

Similarly, binding of Vitamin D to the human Vitamin D3 Receptor was found to result in the recruitment of a specific complex of D Receptor Interacting Proteins - the DRIP coactivator complex, of which the DRIP205 subunit was later identified to be human MED1.

References

Edit history

Date	Action	Author
2008-11-20	Authored	Caudy M
2008-12-03	Created	Caudy M
2009-05-27	Edited	Caudy M
2009-08-29	Reviewed	Freedman LP
2023-11-28	Modified	Wright A

3 submitted entities found in this pathway, mapping to 7 Reactome entities

Input	UniProt Id	Input	UniProt Id	Input	UniProt Id
ENSG00000012504	Q96RI1-1, Q96RI1-2, Q96RI1-3, Q96RI1-4	ENSG00000119508	Q92570-1, Q92570-2	ENSG00000123358	P22736

10. Drug resistance of ALK mutants (R-HSA-9700649)

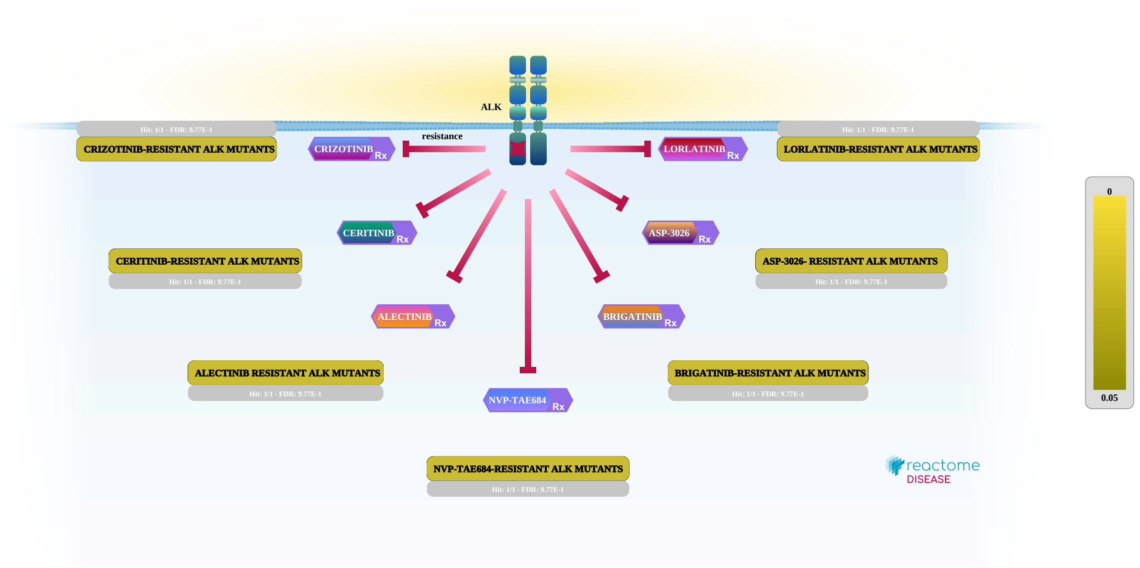

Diseases: cancer.

Aberrant ALK activity arises through fusions, point mutations, overexpression or amplifications and has been shown to be an oncogenic driver in a number of cancers including anaplastic large cell lymphoma (ALCL), non-small cell lung cancer (NSCLC), inflammatory myofibroblastic tumors (IMTs) neuroblastomas and more (reviewed in Della Corte et al, 2018; Lin et al, 2017). As a result, ALK is a promising therapeutic target for inhibition with tyrosine kinase inhibitors. Crizotinib, ceritinib, brigatinib, alectinib and lorlatinib are all approved for the treatment of ALK-driven cancers, however resistance commonly develops either as a result of accumulating secondary mutations, or through activation of bypass pathways that remove the dependence on ALK signaling (reviewed in Della Corte et al, 2017; Roskoski, 2013; Lin et al, 2017).

References

Troiani T, Viscardi G, Morgillo F, Fasano M, Martinelli E, Ciardiello F, ... Di Liello R (2018). Role and targeting of anaplastic lymphoma kinase in cancer. Mol. Cancer, 17, 30.

Roskoski R (2013). Anaplastic lymphoma kinase (ALK): structure, oncogenic activation, and pharmacological inhibition. Pharmacol. Res., 68, 68-94.

Shaw AT, Riely GJ & Lin JJ (2017). Targeting ALK: Precision Medicine Takes on Drug Resistance. Cancer Discov, 7, 137-155.

Edit history

Date	Action	Author
2020-09-16	Created	Rothfels K
2021-03-22	Authored	Rothfels K
2021-03-30	Edited	Rothfels K
2021-05-04	Reviewed	Inghirami G
2023-10-12	Modified	Weiser JD

1 submitted entities found in this pathway, mapping to 1 Reactome entities

Input	UniProt Id
ENSG00000171094	Q9UM73

11. NVP-TAE684-resistant ALK mutants (R-HSA-9717301)

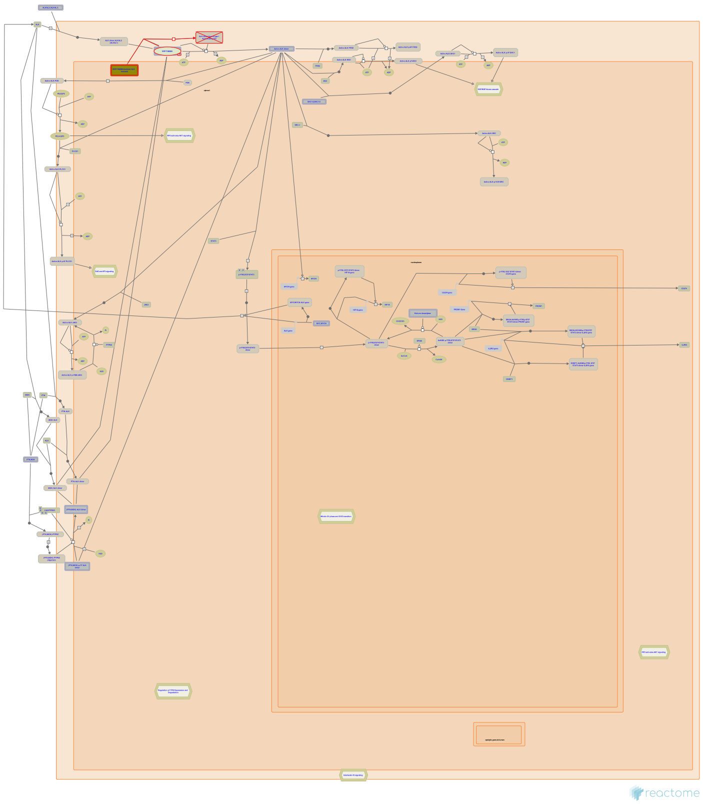

Cellular compartments: plasma membrane, cytosol.

Diseases: cancer.

NVP TAE684 is a second generation tyrosine kinase inhibitor with activity against some ALK mutants, including some that show resistance to crizotinib (George et al, 2008; Sasaki et al, 2011; Heuckmann et al, 2011; Ceccon et al, 2013). This pathway describes ALK mutants that show resistance to inhibition by NVP TAE684.

References

Lovly CM, Grütter C, Heynck S, Sos ML, Thomas RK, Peifer M, ... Hölzel M (2011). ALK mutations conferring differential resistance to structurally diverse ALK inhibitors. Clin. Cancer Res., 17, 7394-401.

Zozulya S, George RE, Gilliland DG, Morris SW, Luther W, London WB, ... Xue L (2008). Activating mutations in ALK provide a therapeutic target in neuroblastoma. Nature, 455, 975-8.

Ceccon M, Mologni L, Scapozza L, Bisson W & Gambacorti-Passerini C (2013). Crizotinib-resistant NPM-ALK mutants confer differential sensitivity to unrelated Alk inhibitors. Mol. Cancer Res., 11, 122-32.

Zheng W, Lindeman N, Capelletti M, Gray NS, Wong KK, Christensen JG, ... Du J (2011). A novel ALK secondary mutation and EGFR signaling cause resistance to ALK kinase inhibitors. Cancer Res., 71, 6051-60.

Edit history

Date	Action	Author
2021-03-08	Created	Rothfels K

Date	Action	Author
2021-03-22	Authored	Rothfels K
2021-03-30	Edited	Rothfels K
2021-05-04	Reviewed	Inghirami G
2023-03-08	Modified	Matthews L

1 submitted entities found in this pathway, mapping to 1 Reactome entities

Input	UniProt Id
ENSG00000171094	Q9UM73

12. ceritinib-resistant ALK mutants (R-HSA-9717323)

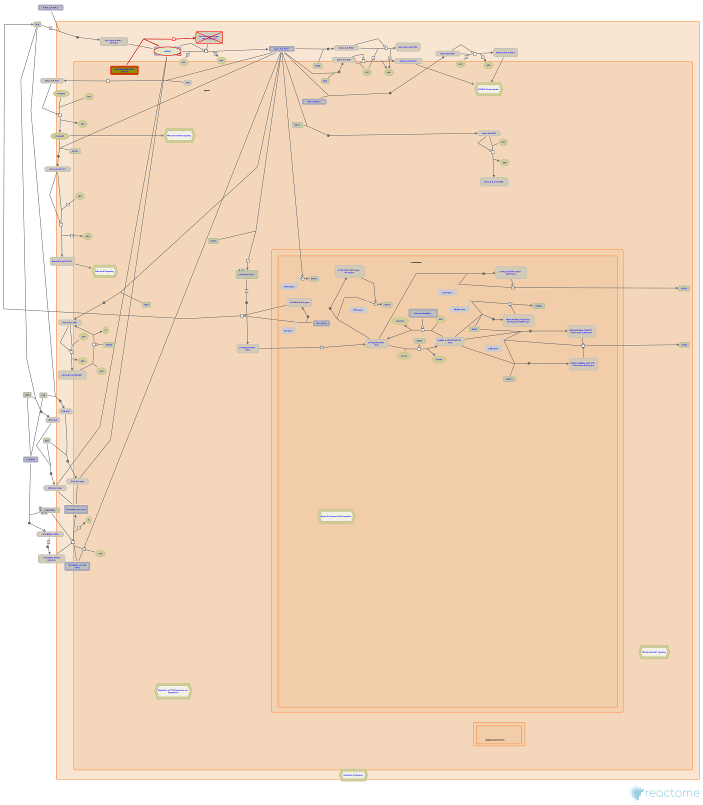

Cellular compartments: plasma membrane, cytosol.

Diseases: cancer.

Ceritinib is a type I TKI that is effective against ALK driven cancers and is approved for treatment of NSCLC. Ceritinib is a second-generation TKI that shows activity against a number of crizotinib-resistant ALK alleles, however, resistance to ceritinib has also been documented. This pathway describes ALK mutants that are resistant to inhibition with ceritinib (reviewed in Lovly and Pao, 2012; Lin et al, 2017; Della Corte et al, 2018).

References

Troiani T, Viscardi G, Morgillo F, Fasano M, Martinelli E, Ciardiello F, ... Di Liello R (2018). Role and targeting of anaplastic lymphoma kinase in cancer. Mol. Cancer, 17, 30.

Lovly CM & Pao W (2012). Escaping ALK inhibition: mechanisms of and strategies to overcome resistance. Sci Transl Med, 4, 120ps2.

Shaw AT, Riely GJ & Lin JJ (2017). Targeting ALK: Precision Medicine Takes on Drug Resistance. Cancer Discov, 7, 137-155.

Edit history

Date	Action	Author
2021-03-08	Created	Rothfels K
2021-03-22	Authored	Rothfels K
2021-03-30	Edited	Rothfels K
2021-05-04	Reviewed	Inghirami G

Date	Action	Author
2023-03-08	Modified	Matthews L

1 submitted entities found in this pathway, mapping to 1 Reactome entities

Input	UniProt Id
ENSG00000171094	Q9UM73

13. lorlatinib-resistant ALK mutants (R-HSA-9717329)

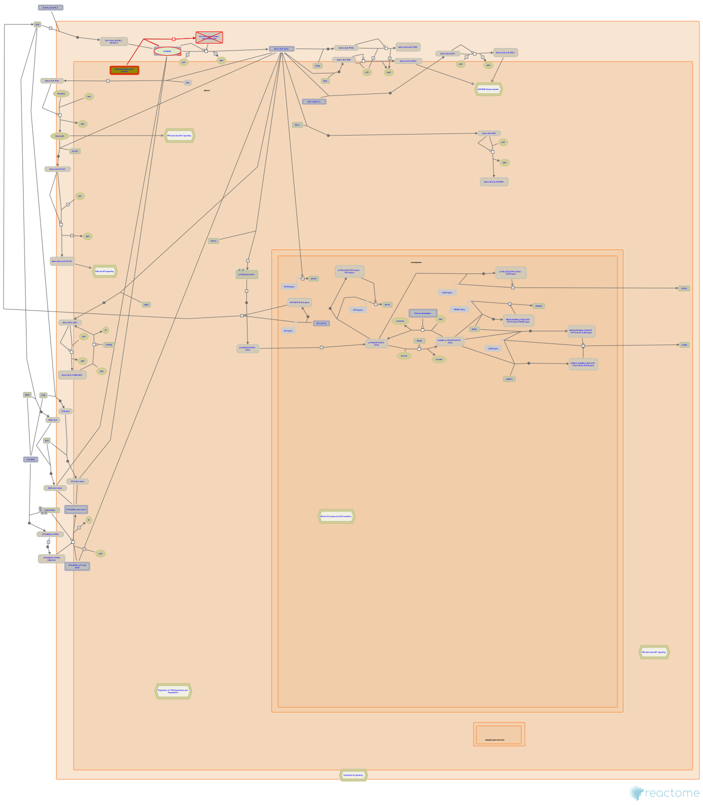

Cellular compartments: plasma membrane, cytosol.

Diseases: cancer.

Lorlatinib is a third generation tyrosine kinase inhibitor with effectiveness against ALK and ROS rearranged cancers. This pathway describes ALK mutants that are resistant to inhibition by lorlatinib (Yoda et al, 2018; Takahashi et al, 2020; reviewed in Della Corte et al, 2018; Lin et al, 2017; Facchinietti et al, 2016).

References

Bria E, Graziano P, Rossi G, Novello S, Di Maio M, Tiseo M & Facchinetti F (2016). Tackling ALK in non-small cell lung cancer: the role of novel inhibitors. Transl Lung Cancer Res, 5, 301-21.

Uchibori K, Yanagitani N, Tsukahara M, Okubo K, Nishio M, Seto Y, ... Katayama R (2020). Overcoming resistance by ALK compound mutation (I1171S + G1269A) after sequential treatment of multiple ALK inhibitors in non-small cell lung cancer. Thorac Cancer, 11, 581-587.

Troiani T, Viscardi G, Morgillo F, Fasano M, Martinelli E, Ciardiello F, ... Di Liello R (2018). Role and targeting of anaplastic lymphoma kinase in cancer. Mol. Cancer, 17, 30.

Mino-Kenudson M, Friboulet L, Benes CH, Engelman JA, Yoda S, Gainor JF, ... Lennerz JK (2018). Sequential ALK Inhibitors Can Select for Lorlatinib-Resistant Compound <i>ALK</i> Mutations in ALK-Positive Lung Cancer. Cancer Discov, 8, 714-729.

Shaw AT, Riely GJ & Lin JJ (2017). Targeting ALK: Precision Medicine Takes on Drug Resistance. Cancer Discov, 7, 137-155.

Edit history

Date	Action	Author
2021-03-08	Created	Rothfels K
2021-03-22	Authored	Rothfels K
2021-03-30	Edited	Rothfels K
2021-05-04	Reviewed	Inghirami G
2023-03-08	Modified	Matthews L

1 submitted entities found in this pathway, mapping to 1 Reactome entities

Input	UniProt Id
ENSG00000171094	Q9UM73

14. ASP-3026-resistant ALK mutants (R-HSA-9717264)

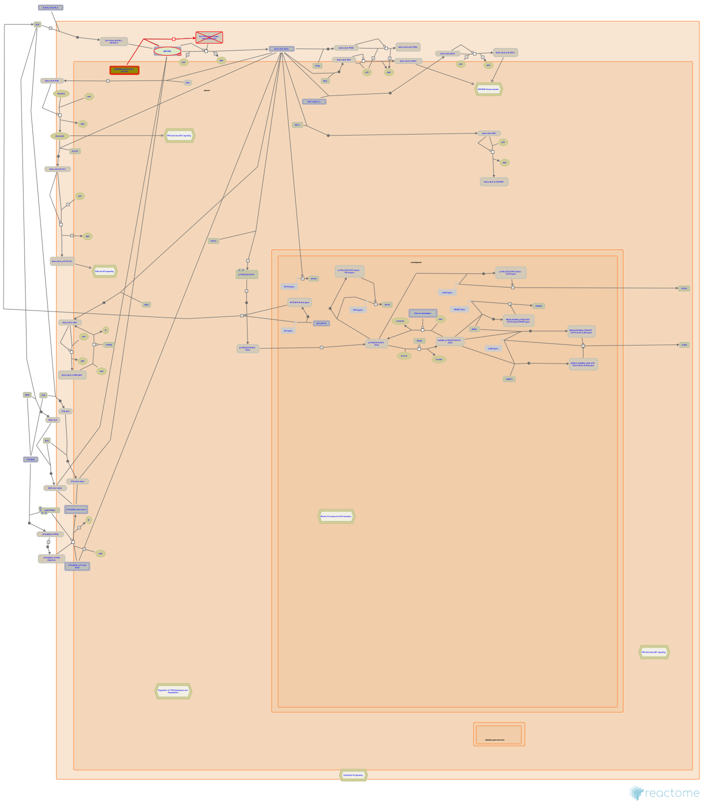

Cellular compartments: plasma membrane, cytosol.

Diseases: cancer.

ASP3026 is a second generation tyrosine kinase inhibitor with activity against ALK fusions in non-small cell lung cancers (NSCLC) and anaplastic large cell lymphomas (ALCLs). This pathway describes ALK mutants that are resistant to inhibition by ASP3026 (Amin et al, 2016; Katayama et al, 2014; George et al, 2008; Mori et al, 2014; reviewed Roskoski, 2013; Lovly and Pao, 2012)

References

Okuno Y, Iafrate AJ, Friboulet L, Koike S, Shaw AT, Engelman JA, ... Katayama R (2014). Two novel ALK mutations mediate acquired resistance to the next-generation ALK inhibitor alectinib. Clin Cancer Res, 20, 5686-96.

Zozulya S, George RE, Gilliland DG, Morris SW, Luther W, London WB, ... Xue L (2008). Activating mutations in ALK provide a therapeutic target in neuroblastoma. Nature, 455, 975-8.

Gokhale V, Groysman MJ, Pongtornpipat P, Wang M, Tapia EO, Rajan SS, ... Li L (2016). TKI sensitivity patterns of novel kinase-domain mutations suggest therapeutic opportunities for patients with resistant ALK+ tumors. Oncotarget, 7, 23715-29.

Lovly CM & Pao W (2012). Escaping ALK inhibition: mechanisms of and strategies to overcome resistance. Sci Transl Med, 4, 120ps2.

Roskoski R (2013). Anaplastic lymphoma kinase (ALK): structure, oncogenic activation, and pharmacological inhibition. Pharmacol. Res., 68, 68-94.

Edit history

Date	Action	Author
2021-03-08	Created	Rothfels K
2021-03-22	Authored	Rothfels K
2021-03-30	Edited	Rothfels K
2021-05-04	Reviewed	Inghirami G
2023-03-08	Modified	Matthews L

1 submitted entities found in this pathway, mapping to 1 Reactome entities

Input	UniProt Id
ENSG00000171094	Q9UM73

15. alectinib-resistant ALK mutants (R-HSA-9717316)

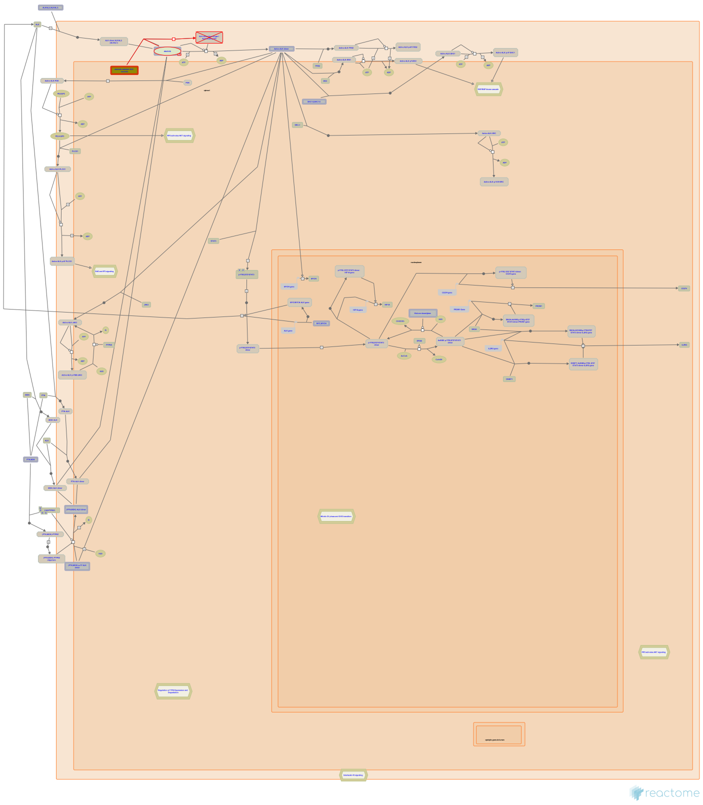

Cellular compartments: plasma membrane, cytosol.

Diseases: cancer.

Alectinib is a second generation tyrosine kinase inhibitor that is approved for use in ALK positive non-small cell lung cancers (NSCLCs). Alectinib is effective against a number of ALK mutants that arise after treatment with crizotinib, however resistance to alectinib has also been reported. This pathway describes ALK mutants that are resistant to inhibition with alectinib (reviewed in Lovly and Pao, 2012; Lin et al, 2017; Della Corte et al, 2018; Hallberg and Palmer, 2016).

References

Troiani T, Viscardi G, Morgillo F, Fasano M, Martinelli E, Ciardiello F, ... Di Liello R (2018). Role and targeting of anaplastic lymphoma kinase in cancer. Mol. Cancer, 17, 30.

Lovly CM & Pao W (2012). Escaping ALK inhibition: mechanisms of and strategies to overcome resistance. Sci Transl Med, 4, 120ps2.

Shaw AT, Riely GJ & Lin JJ (2017). Targeting ALK: Precision Medicine Takes on Drug Resistance. Cancer Discov, 7, 137-155.

Palmer RH & Hallberg B (2013). Mechanistic insight into ALK receptor tyrosine kinase in human cancer biology. Nat. Rev. Cancer, 13, 685-700.

Edit history

Date	Action	Author
2021-03-08	Created	Rothfels K
2021-03-22	Authored	Rothfels K

Date	Action	Author
2021-03-30	Edited	Rothfels K
2021-05-04	Reviewed	Inghirami G
2023-03-08	Modified	Matthews L

1 submitted entities found in this pathway, mapping to 1 Reactome entities

Input	UniProt Id
ENSG00000171094	Q9UM73

16. brigatinib-resistant ALK mutants (R-HSA-9717319)

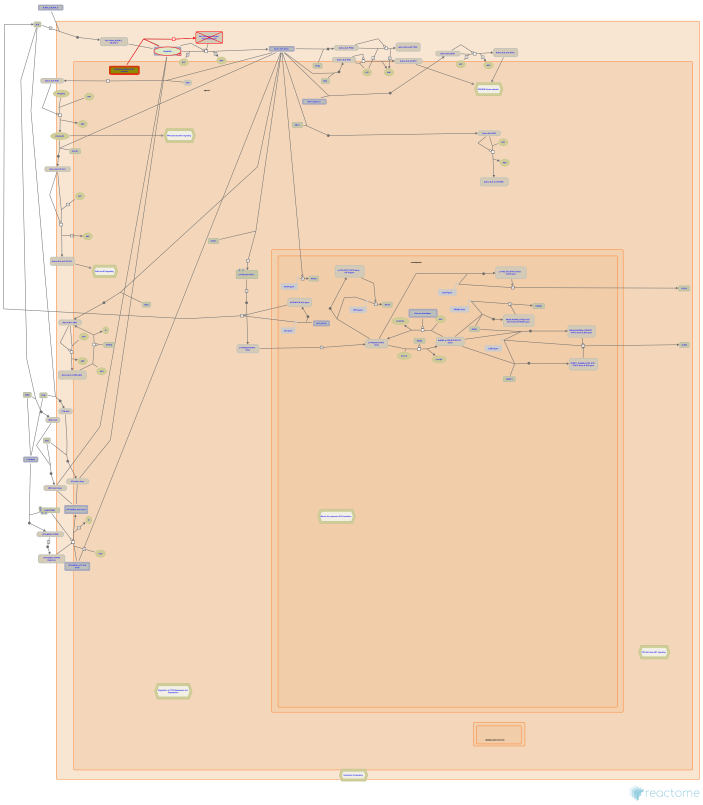

Cellular compartments: plasma membrane, cytosol.

Diseases: cancer.

Brigatinib is a second generation tyrosine kinase inhibitor with activity against ALK. This pathway describes ALK mutants that are resistant to inhibition by brigatinib (reviewed in Della Corte et al, 2018; Roskoski, 2013; Lin and Pao, 2017; Hallberg and Palmer, 2013).

References

Troiani T, Viscardi G, Morgillo F, Fasano M, Martinelli E, Ciardiello F, ... Di Liello R (2018). Role and targeting of anaplastic lymphoma kinase in cancer. Mol. Cancer, 17, 30.

Roskoski R (2013). Anaplastic lymphoma kinase (ALK): structure, oncogenic activation, and pharmacological inhibition. Pharmacol. Res., 68, 68-94.

Shaw AT, Riely GJ & Lin JJ (2017). Targeting ALK: Precision Medicine Takes on Drug Resistance. Cancer Discov, 7, 137-155.

Palmer RH & Hallberg B (2013). Mechanistic insight into ALK receptor tyrosine kinase in human cancer biology. Nat. Rev. Cancer, 13, 685-700.

Edit history

Date	Action	Author
2021-03-08	Created	Rothfels K
2021-03-22	Authored	Rothfels K
2021-03-30	Edited	Rothfels K

Date	Action	Author
2021-05-04	Reviewed	Inghirami G
2023-03-08	Modified	Matthews L

1 submitted entities found in this pathway, mapping to 1 Reactome entities

Input	UniProt Id
ENSG00000171094	Q9UM73

17. crizotinib-resistant ALK mutants (R-HSA-9717326)

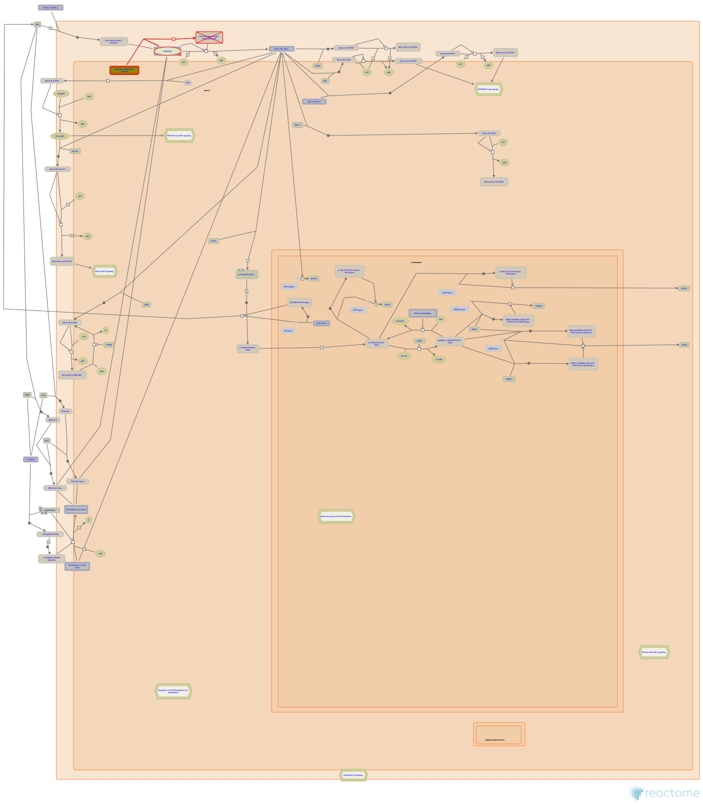

Cellular compartments: plasma membrane, cytosol.

Diseases: cancer.

Crizotinib is a type I tyrosine kinase inhibitor that is approved for treatment of ALK-positive non-small cell lung cancer. Crizotinib is also effective against ALCL and IMTs. Development of resistance to crizotinib is not uncommon, however, with patients acquiring secondary mutations or amplifications of the ALK gene that limit the effectiveness of the drug. This pathway describes ALK mutants that are resistant to crizotinib-mediated inhibition (reviewed in Roskoski, 2013; Lin et al, 2017; Della Corte et al, 2018).

References

Troiani T, Viscardi G, Morgillo F, Fasano M, Martinelli E, Ciardiello F, ... Di Liello R (2018). Role and targeting of anaplastic lymphoma kinase in cancer. Mol. Cancer, 17, 30.

Roskoski R (2013). Anaplastic lymphoma kinase (ALK): structure, oncogenic activation, and pharmacological inhibition. Pharmacol. Res., 68, 68-94.

Shaw AT, Riely GJ & Lin JJ (2017). Targeting ALK: Precision Medicine Takes on Drug Resistance. Cancer Discov, 7, 137-155.

Edit history

Date	Action	Author
2021-03-08	Created	Rothfels K
2021-03-22	Authored	Rothfels K
2021-03-30	Edited	Rothfels K

Date	Action	Author
2021-05-04	Reviewed	Inghirami G
2023-10-12	Modified	Weiser JD

1 submitted entities found in this pathway, mapping to 1 Reactome entities

Input	UniProt Id
ENSG00000171094	Q9UM73

18. Hereditary fructose intolerance (R-HSA-5657560)

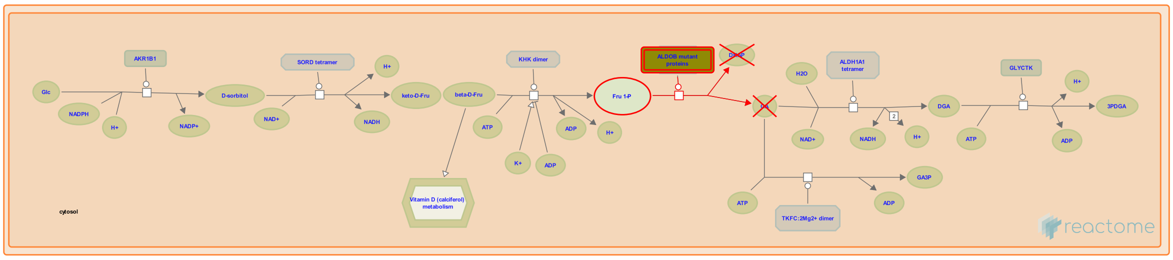

Diseases: hereditary fructose intolerance syndrome.

Deficiencies in aldolase B arising from mutations in the aldolase B gene (ALDOB) prevent the cleavage of fructose 1-phosphate to glyceraldehyde (GA) and dihydroxyacetone phosphate (DHAP), leading to hereditary fructose intolerance (HFI). This autosomal recessive disorder is potentially fatal, but can be managed by exclusion of fructose from the diet (Cox et al. 1988; Tolan 1995).

References

Tolan DR (1995). Molecular basis of hereditary fructose intolerance: mutations and polymorphisms in the human aldolase B gene. Hum Mutat, 6, 210-8.

Cross NC, Cox TM & Tolan DR (1988). Catalytic deficiency of human aldolase B in hereditary fructose intolerance caused by a common missense mutation. Cell, 53, 881-5.

Edit history

Date	Action	Author
2014-12-13	Created	D'Eustachio P
2015-01-29	Edited	D'Eustachio P
2015-01-29	Reviewed	Jassal B
2015-01-29	Authored	D'Eustachio P
2015-02-17	Reviewed	Tolan DR, Timson DJ
2023-10-12	Modified	Weiser JD

1 submitted entities found in this pathway, mapping to 1 Reactome entities

Input	UniProt Id
ENSG00000136872	P05062

19. Laminin interactions (R-HSA-3000157)

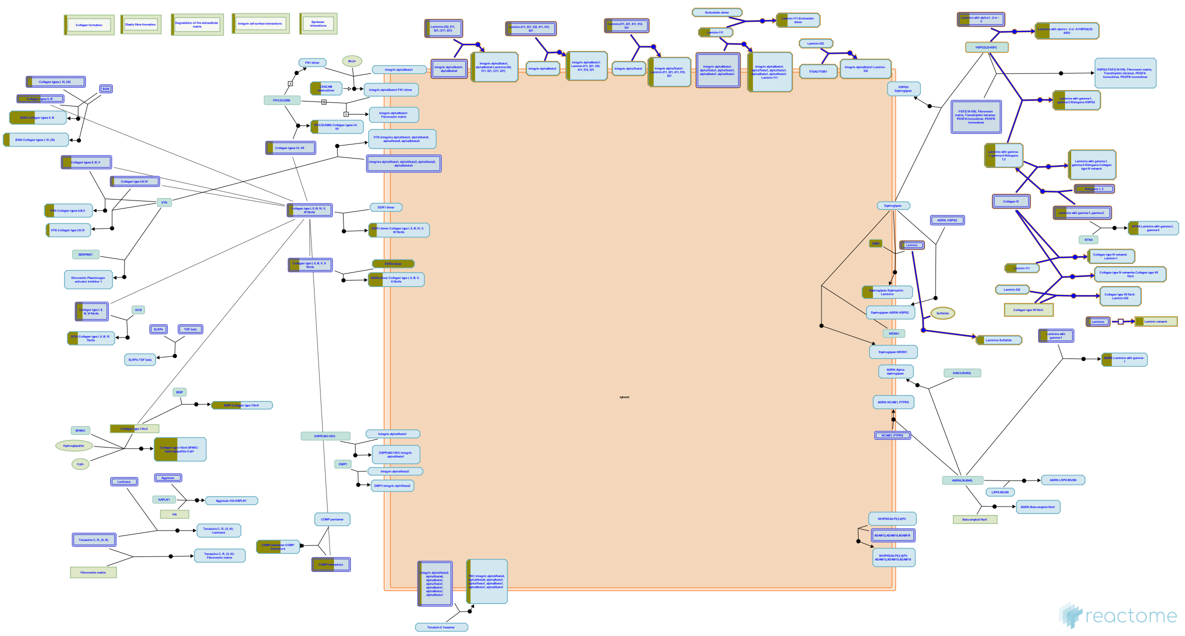

Laminins are a large family of conserved, multidomain trimeric basement membrane proteins. There are many theoretical trimer combinations but only 18 have been described (Domogatskaya et al. 2012, Miner 2008, Macdonald et al. 2010) and the existence of isoforms laminin-212 and/or laminin-222 (Durbeej et al. 2010) awaits further confirmation. The chains assemble through coiled-coil domains at their C-terminal end. Alpha chains additionally have a large C-terminal globular domain containing five LG subdomains (LG1-5). The N termini are often referred to as the short arms. These have varying numbers of laminin-type epidermal growth factor-like (LE) repeats. Trimer assembly is controlled by highly specific coiled-coil interactions (Domogatskaya et al. 2012). Some laminin isoforms are modified extracellularly by proteolytic processing at the N- or C-terminal ends prior to their binding to cellular receptors or other matrix molecules (Tzu & Marinkovitch 2008).

The cell adhesion properties of laminins are mediated primarily through the alpha chain G domain to integrins, dystroglycan, Lutheran glycoprotein, or sulfated glycolipids. The N-terminal globular domains of the alpha-1 (Colognato-Pyke et al. 1995) and alpha-2 chains (Colognato et al. 1997) and globular domains VI (Nielsen & Yamada 2001) and IVa (Sasaki & Timpl 2001) of the alpha-5 chain can bind to several integrin isoforms (alpha1beta1, alpha2beta1, alpha3beta1, and alphaVbeta3), which enables cell binding at both ends of laminins with these alpha chains.

References

Domogatskaya A, Rodin S & Tryggvason K (2012). Functional diversity of laminins. Annu. Rev. Cell Dev. Biol., 28, 523-53.

Edit history

Date	Action	Author
2008-05-07	Reviewed	Hynes R, Humphries MJ, Yamada KM
2012-08-08	Authored	Jupe S
2013-01-24	Created	Jupe S
2013-08-13	Edited	Jupe S

Date	Action	Author
2013-08-13	Reviewed	Ricard-Blum S
2023-11-16	Modified	Wright A

3 submitted entities found in this pathway, mapping to 3 Reactome entities

Input	UniProt Id	Input	UniProt Id	Input	UniProt Id
ENSG00000087303	Q14112	ENSG00000101680	P25391	ENSG00000196569	P24043

20. Defective SLC26A3 causes congenital secretory chloride diarrhea 1 (DIAR1) (R-HSA-5619085)

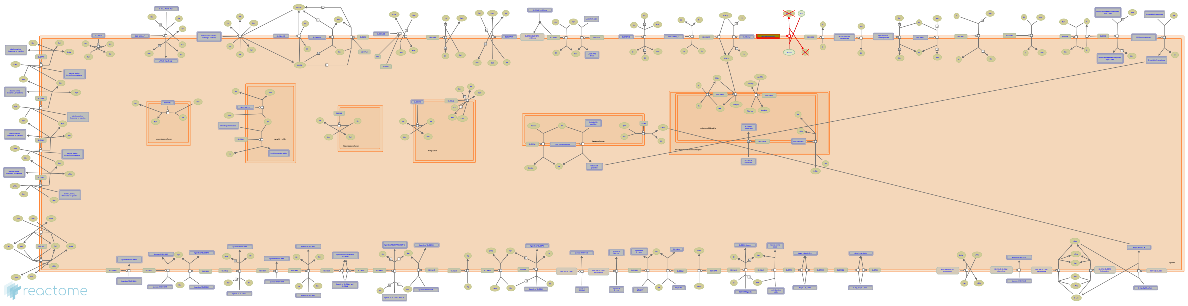

Diseases: secretory diarrhea.

Solute carrier (SLC) genes that code chloride (Cl-)/bicarbonate (HCO3-) exchanger proteins are the SLC4 and SLC26 families. The chloride anion exchanger SLC26A3 (aka down-regulated in adenoma, DRA) mediates electrolyte and fluid absorption in the colon. It is also localised to the midpiece tail membrane of sperm where it plays a role in Cl-/HCO3- homeostasis during sperm epididymal maturation. Defects in SLC26A3 cause congenital chloride diarrhea 1 (DIAR1), a disease characterised by watery stools containing an excess of chloride resulting in dehydration, hypokalemia, and metabolic alkalosis (Alper & Sharma 2013, Wedenoja et al. 2011).

References

Alper SL & Sharma AK (2013). The SLC26 gene family of anion transporters and channels. Mol. Aspects Med., 34, 494-515.

Kere J, Wedenoja S, Pekansaari E, Mäkelä S, Holmberg C & Höglund P (2011). Update on SLC26A3 mutations in congenital chloride diarrhea. Hum. Mutat., 32, 715-22.

Edit history

Date	Action	Author
2014-08-22	Edited	Jassal B
2014-08-22	Authored	Jassal B
2014-08-22	Created	Jassal B
2015-08-04	Reviewed	Broer S
2023-11-28	Modified	Wright A

1 submitted entities found in this pathway, mapping to 1 Reactome entities

Input	UniProt Id
ENSG00000091138	P40879

21. Defective SLC17A8 causes autosomal dominant deafness 25 (DFNA25) (R-HSA-5619076)

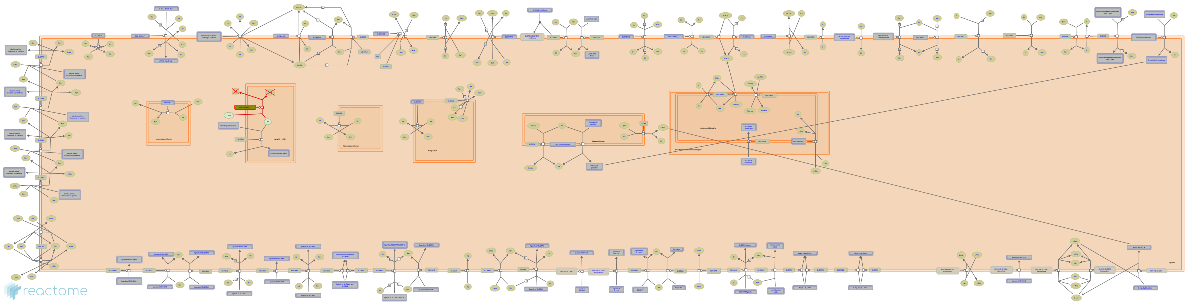

Diseases: autosomal dominant nonsyndromic deafness.

There are two classes of glutamate transporters; the excitatory amino acid transporters (EAATs) which depend on an electrochemical gradient of Na+ ions and vesicular glutamate transporters (VGLUTs) which are proton-dependent. Together, these transporters uptake and release glutamate to mediate this neurotransmitter's excitatory signal and are part of the glutamate-glutamine cycle. Three members of the SLC17A gene family (7, 6 and 8) encode VGLUTs 1-3 respectively. This uptake is thought to be coupled to the proton electrochemical gradient generated by the vacuolar type H+-ATPase. They are all expressed in the CNS in neuron-rich areas but SLC17A8 (VGLUT3) is also expressed on astrocytes and in the liver and kidney. Defects in SLC17A8 can cause autosomal dominant deafness 25 (DFNA25; MIM:605583), a form of non-syndromic sensorineural hearing loss. The cochlea expresses SLC17A8 and in mice which lack this transporter are congenitally deaf. Hearing loss is due to the lack of glutamate release by inner hair cells therefore a loss of synaptic transmission at the IHC-afferent nerve synapse. Successful restoration of hearing by gene replacement in mice could be a significant advance toward gene therapy of human deafness (Ruel et al. 2008, Akil et al. 2012).

References

During M, Burke K, Lustig LR, Edwards RH, Seal RP, Akil O, ... Wang C (2012). Restoration of hearing in the VGLUT3 knockout mouse using virally mediated gene therapy. Neuron, 75, 283-93.

Smith RJ, El Mestikawy S, Bersot T, Nouvian R, Giros B, Ruel J, ... Van Rybroek JM (2008). Impairment of SLC17A8 encoding vesicular glutamate transporter-3, VGLUT3, underlies nonsyndromic deafness DFNA25 and inner hair cell dysfunction in null mice. Am. J. Hum. Genet., 83, 278-92.

Edit history

Date	Action	Author
2014-08-22	Edited	Jassal B
2014-08-22	Authored	Jassal B
2014-08-22	Created	Jassal B
2015-08-04	Reviewed	Broer S
2023-10-12	Modified	Weiser JD

1 submitted entities found in this pathway, mapping to 1 Reactome entities

Input	UniProt Id
ENSG00000179520	Q8NDX2

22. Defective ABCC9 causes CMD10, ATFB12 and Cantu syndrome (R-HSA-5678420)

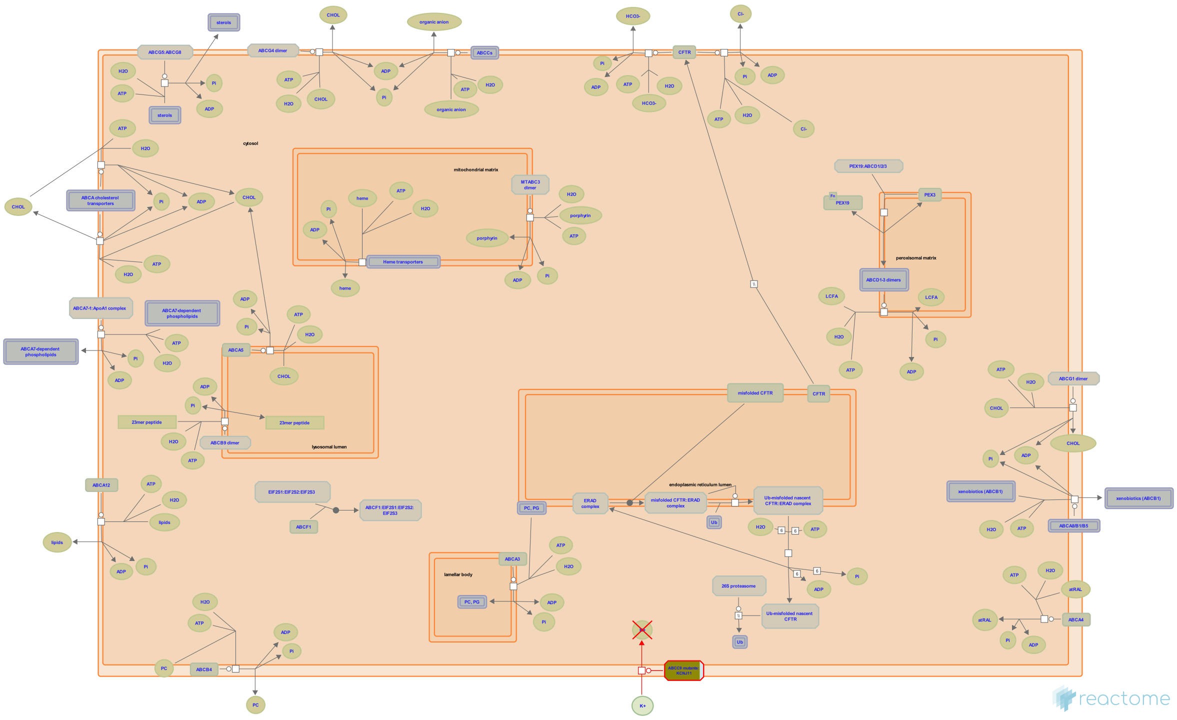

Diseases: hypertrichosis, familial atrial fibrillation, osteochondrodysplasia, dilated cardiomyopathy.

ATP-binding cassette sub-family C member 9 (ABCC9) forms cardiac and smooth muscle-type KATP channels with ATP-sensitive inward rectifier potassium channel 11 (KCNJ11). KCNJ11 forms the channel pore while ABCC9 is required for activation and regulation (Babenko et al. 1998, Tammaro & Ashcroft 2007). Inward rectifier potassium channels favor the flow of potassium into the cell rather than out of it. KATP channels open and close in response to intracellular changes in the ADP/ATP ratio, thereby linking the metabolic state of the cell to its membrane potential. Inhibition of KATP channel activity causes membrane depolarization and thereby activation of voltage-dependent Ca2+ channels, leading to Ca2+ influx and a rise in intracellular Ca2+ concentration. Correct maintenance of calcium balance is essential for the normal functioning of the heart.

Defects in ABCC9 can cause dilated cardiomyopathy 10 (CMD10: MIM:608569), a disorder characterised by ventricular dilation and impaired systolic function, resulting in congestive heart failure and arrhythmia (Bienengraeber et al. 2004). Defects in ABCC9 can also cause familial atrial fibrillation 12 (ATFB12; MIM:614050), characterised by disorganized atrial electrical activity and ineffective atrial contraction resulting in blood stasis in the atria and reduces ventricular filling. It can result in palpitations, syncope, thromboembolic stroke, and congestive heart failure (Olson et al. 2007). Defects in ABCC9 can also cause hypertrichotic osteochondrodysplasia (Cantu syndrome; MIM:239850), a rare disorder characterised by congenital hypertrichosis, neonatal macrosomia, a distinct osteochondrodysplasia and cardiomegaly (van Bon et al. 2012, Harakalova et al. 2012).

References

van Lieshout S, van Haelst MM, Amor DJ, Asselbergs FW, Terhal PA, van der Smagt JJ, ... van Haaften G (2012). Dominant missense mutations in ABCC9 cause Cantú syndrome. Nat. Genet., 44, 793-6.

Brunner HG, Isidor B, van Bon BW, Grange DK, Morava E, Eser M, ... Wieskamp N (2012). Cantú syndrome is caused by mutations in ABCC9. Am. J. Hum. Genet., 90, 1094-101.

Ashcroft FM & Tammaro P (2007). A mutation in the ATP-binding site of the Kir6.2 subunit of the KATP channel alters coupling with the SUR2A subunit. J. Physiol. (Lond.), 584, 743-53.

Moreau C, Asirvatham SJ, Seino S, Olson TM, Liu XK, Zingman LV, ... Alekseev AE (2007). KATP channel mutation confers risk for vein of Marshall adrenergic atrial fibrillation. Nat Clin Pract Cardiovasc Med, 4, 110-6.

Bryan J, Gonzalez G, Aguilar-Bryan L & Babenko AP (1998). Reconstituted human cardiac KATP channels: functional identity with the native channels from the sarcolemma of human ventricular cells. Circ. Res., 83, 1132-43.

Edit history

Date	Action	Author
2015-02-24	Edited	Jassal B
2015-02-24	Authored	Jassal B
2015-02-24	Created	Jassal B
2015-04-28	Reviewed	Moitra K
2023-10-12	Modified	Weiser JD

1 submitted entities found in this pathway, mapping to 1 Reactome entities

Input	UniProt Id
ENSG00000069431	O60706

23. Defective SLC34A2 causes pulmonary alveolar microlithiasis (PALM) (R-HSA-5619045)

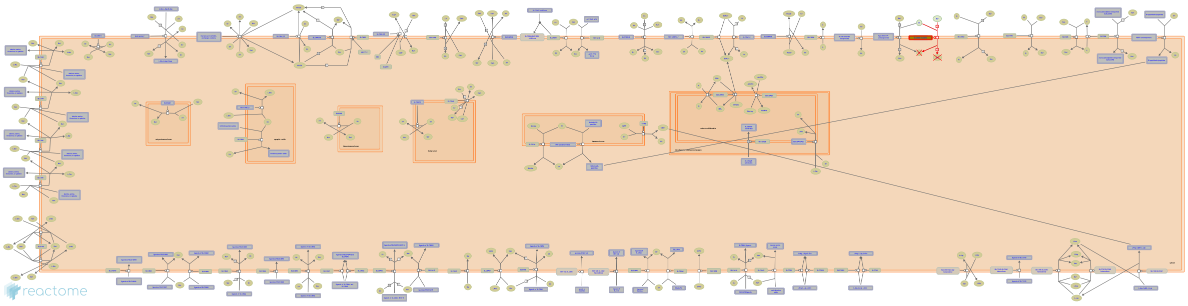

Diseases: pulmonary alveolar microlithiasis.

SLC34A1 and 2 encode Na+/Pi cotransporters, which cotransport divalent phosphate (PO4(2-), Pi) with 3 Na+ ions. SLC34A2 is abundantly expressed in lung and to a lesser extent in tissues of epithelial origin including small intestine, pancreas, prostate, and kidney. Defects in SLC34A2 are a cause of pulmonary alveolar microlithiasis (PALM; MIM:265100), a rare disease characterised by the deposition of calcium phosphate microliths throughout the lungs. The disease follows a long-term progressive course, resulting in a slow deterioration of lung function (Corut et al. 2006, Forster et al. 2013).

References

Corut A, Tolun A, Yildirim Z, Gocmen A, Senyigit A, Ozcelik U, ... Ugur SA (2006). Mutations in SLC34A2 cause pulmonary alveolar microlithiasis and are possibly associated with testicular microlithiasis. Am J Hum Genet, 79, 650-6.

Biber J, Forster IC, Hernando N & Murer H (2013). Phosphate transporters of the SLC20 and SLC34 families. Mol. Aspects Med., 34, 386-95.

Edit history

Date	Action	Author
2014-08-22	Edited	Jassal B
2014-08-22	Authored	Jassal B
2014-08-22	Created	Jassal B
2015-08-04	Reviewed	Broer S
2023-11-28	Modified	Wright A

1 submitted entities found in this pathway, mapping to 1 Reactome entities

Input	UniProt Id
ENSG00000157765	O95436

24. Defective VWF binding to collagen type I (R-HSA-9845622)

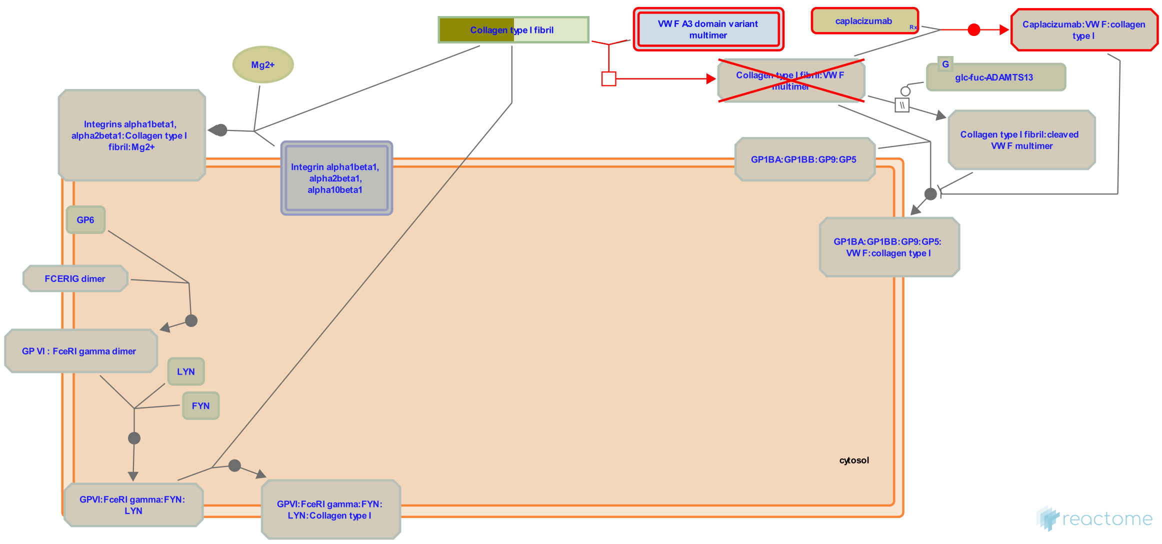

Diseases: blood platelet disease.

Upon vascular injury, circulating von Willebrand factor (VWF) binds to exposed vascular collagen. This Reactome event shows defective binding of VWF to collagen type I caused by loss-of-function mutations in the A3 domain of VWF found in patients with von Willebrand disease (VWD) type 2M, which is characterized by defects in platelet adhesion and/or collagen binding with normal or sub-normal VWF multimer distribution.

References

Nieswandt B, Stegner D, Mewburn J, Brown C, Vidal B, Lillicrap D, ... Crawford B (2014). Analysis of the role of von Willebrand factor, platelet glycoprotein VI-, and �2�1-mediated collagen binding in thrombus formation. Blood, 124, 1799-807.

Gill S, McKinnon TA, Mellars G, Sutherland M, Riddell AF, Brown SA, ... Millar CM (2009). Characterization of W1745C and S1783A: 2 novel mutations causing defective collagen binding in the A3 domain of von Willebrand factor. Blood, 114, 3489-96.

Montgomery RR, Christopherson PA, Friedman KD, Hoffmann RG, Flood VH, Lederman CA & Wren JS (2010). Absent collagen binding in a VWF A3 domain mutant: utility of the VWF:CB in diagnosis of VWD. J Thromb Haemost, 8, 1431-3.

Simons A, Brons PPT, Blijlevens NMA, Meijer D, Nieuwenhuizen L, Schols SEM, ... Maas DPMSM (2022). Von Willebrand disease type 2M: Correlation between genotype and phenotype. J Thromb Haemost, 20, 316-327.

Edit history

Date	Action	Author
2023-01-07	Authored	Shamovsky V
2023-10-06	Created	Shamovsky V
2023-11-06	Reviewed	Gao R
2023-11-07	Modified	Shamovsky V
2023-11-07	Edited	Shamovsky V

1 submitted entities found in this pathway, mapping to 1 Reactome entities

Input	UniProt Id
ENSG00000108821	P02452

25. Defective SLC34A2 causes PALM (R-HSA-5687583)

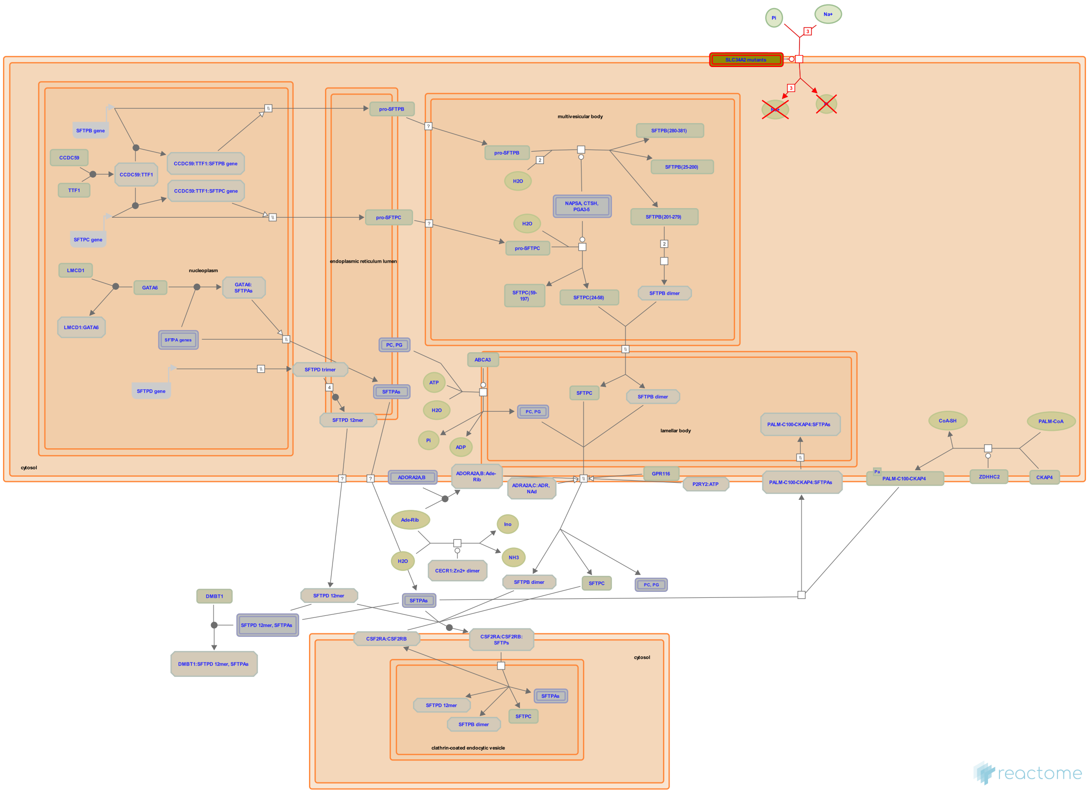

Diseases: pulmonary alveolar microlithiasis.

The human gene SLC34A2 encodes NaPi-2b which is abundantly expressed in lung and to a lesser degree in epithelia of other tissues including small intestine, pancreas, prostate, and kidney. In the lung, SLC34A2 is expressed only in alveolar type II cells, which are responsible for surfactant production, so it is proposed that it uptakes liberated phosphate from the alveolar fluid for surfactant production. SLC34A2 cotransports divalent phosphate (HPO4(2-)) with three Na+ ions (electrogenic transport) from the extracellular region into alveolar type II cells. Defects in SLC34A2 can cause pulmonary alveolar microlithiasis (PALM; MIM:265100), a rare disease characterised by the deposition of calcium phosphate microliths (tiny, roundish corpuscles) throughout the lung. Most patients remain asymptomatic for years or decades, the disease following a long-term, progressive course resulting in slow deterioration of lung functions. PALM can result in a potentially lethal disease (Yin et al. 2013, Ferreira Francisco et al. 2013, Whitsett et al. 2015).

References

Shao J, Wu D, Zhao G, Wang H, Dai Y & Yin X (2013). SLC34A2 Gene mutation of pulmonary alveolar microlithiasis: report of four cases and review of literatures. Respir Med, 107, 217-22.

Whitsett JA, Weaver TE & Wert SE (2015). Diseases of pulmonary surfactant homeostasis. Annu Rev Pathol, 10, 371-93.

Marchiori E, Pereira e Silva JL, Ferreira Francisco FA, Hochhegger B & Zanetti G (2013). Pulmonary alveolar microlithiasis. State-of-the-art review. Respir Med, 107, 1-9.

Edit history

Date	Action	Author
2015-04-08	Edited	Jassal B
2015-04-08	Authored	Jassal B
2015-04-08	Created	Jassal B
2015-08-17	Reviewed	D'Eustachio P
2023-11-28	Modified	Wright A

1 submitted entities found in this pathway, mapping to 1 Reactome entities

Input	UniProt Id
ENSG00000157765	O95436

6. Identifiers found

Below is a list of the input identifiers that have been found or mapped to an equivalent element in Reactome, classified by resource.

189 of the submitted entities were found, mapping to 244 Reactome entities

Input	UniProt Id	Input	UniProt Id	Input	UniProt Id
ENSG00000004139	Q6SZW1-1	ENSG00000006283	O43497	ENSG00000010379	Q9NSD5
ENSG00000011347	O43581	ENSG00000012504	Q96RI1-1, Q96RI1-2, Q96RI1-3, Q96RI1-4	ENSG00000015520	Q9UHC9-2
ENSG00000023839	Q92887	ENSG00000050628	P43115	ENSG00000057468	O15457
ENSG00000057657	O75626	ENSG00000065621	Q9H4Y5	ENSG00000067842	Q16720
ENSG00000069431	O60706	ENSG00000070495	Q6NYC1	ENSG00000073756	P35354
ENSG00000077943	P53708	ENSG00000078579	Q9NP95	ENSG00000080644	P32297
ENSG00000087074	O75807	ENSG00000087076	Q9BPX1	ENSG00000087085	P22303
ENSG00000087237	P11597	ENSG00000087303	Q14112	ENSG00000087494	P12272
ENSG00000090273	Q9Y266	ENSG00000091138	P40879	ENSG00000100292	P09601
ENSG00000101049	Q9HBY8	ENSG00000101144	P18075	ENSG00000101680	P25391
ENSG00000102387	Q5H9L4	ENSG00000102760	Q9H4X1	ENSG00000105227	Q9BXM0, Q9BXM0-1
ENSG00000105499	Q9UP65	ENSG00000105641	Q92911	ENSG00000105642	Q92952
ENSG00000106211	P04792	ENSG00000107242	O14986	ENSG00000108576	P31645
ENSG00000108821	P02452	ENSG00000108830	P52198	ENSG00000109063	P11055
ENSG00000109107	P09972	ENSG00000109846	P02511	ENSG00000110169	P02790
ENSG00000112599	Q9UMX6	ENSG00000113263	Q08881	ENSG00000113739	O76061
ENSG00000113905	P04196	ENSG00000115738	Q02363	ENSG00000116329	P41143
ENSG00000116741	P49795	ENSG00000117281	O95971	ENSG00000117601	P01008
ENSG00000118515	O00141	ENSG00000119508	Q92570-1, Q92570-2	ENSG00000120333	O60783
ENSG00000120438	P17987	ENSG00000120616	Q9H2F5	ENSG00000120694	Q92598
ENSG00000120738	P18146	ENSG00000120885	P10909	ENSG00000120903	Q15822
ENSG00000122877	P11161	ENSG00000123358	P22736	ENSG00000124216	O95863
ENSG00000125740	P53539	ENSG00000128422	Q04695	ENSG00000128604	Q13568
ENSG00000128617	P03999	ENSG00000129596	Q16878	ENSG00000130203	P02649
ENSG00000130766	P58004	ENSG00000131620	Q5XXA6	ENSG00000132002	P25685
ENSG00000132510	O15054	ENSG00000132554	Q8NE09	ENSG00000134115	Q9UQ52
ENSG00000134215	Q9UKW4	ENSG00000134242	Q9Y2R2	ENSG00000134321	Q8WXG1
ENSG00000134962	Q86Z14	ENSG00000135519	Q9ULD8	ENSG00000135773	O14815
ENSG00000136872	P05062	ENSG00000136960	Q13822	ENSG00000138379	O14793
ENSG00000138829	P35556	ENSG00000138942	Q96GF1	ENSG00000139219	P02458
ENSG00000139220	O75334	ENSG00000139287	Q8IWU9	ENSG00000139679	P43657
ENSG00000140873	Q8TE60	ENSG00000142528	Q8WTR7	ENSG00000143199	Q96PN6
ENSG00000143320	P29373	ENSG00000143333	O15492	ENSG00000143882	Q8NEY4
ENSG00000144381	P10809	ENSG00000145192	P02765	ENSG00000146021	Q9UH77
ENSG00000146469	P01282	ENSG00000147655	Q6UXX9	ENSG00000149124	Q6IB77
ENSG00000149257	P50454	ENSG00000149781	Q86UX7	ENSG00000149968	P08254
ENSG00000151929	O95817	ENSG00000152137	Q9UJY1	ENSG00000152670	Q9NQI0
ENSG00000154548	Q8WXF0	ENSG00000154734	Q9UHI8	ENSG00000155380	P53985
ENSG00000155980	Q12840	ENSG00000157551	Q99712	ENSG00000157765	O95436

Input	UniProt Id	Input	UniProt Id	Input	UniProt Id
ENSG00000158296	Q8WWT9	ENSG00000158445	Q14721	ENSG00000159388	P78543
ENSG00000160961	Q96JL9	ENSG00000160963	Q96A83	ENSG00000161270	O60500
ENSG00000161681	Q9Y566	ENSG00000162733	Q16832	ENSG00000162772	P18847
ENSG00000162892	Q13007	ENSG00000162896	P01833	ENSG00000163435	P78545
ENSG00000163602	Q8N488	ENSG00000164070	O95757	ENSG00000164093	Q99697
ENSG00000164683	Q9Y5J3	ENSG00000164776	Q16816	ENSG00000164850	Q99527
ENSG00000164949	P55040	ENSG00000166225	Q8WU20	ENSG00000166391	Q3SYC2
ENSG00000166592	P55042	ENSG00000167280	Q8NFI3	ENSG00000167281	A6NFN3
ENSG00000167306	Q9ULV0	ENSG00000168439	P31948	ENSG00000168539	P11229
ENSG00000168646	Q9Y2T1	ENSG00000169896	P11215	ENSG00000170345	P01100
ENSG00000171094	Q9UM73	ENSG00000171777	Q8TDF6	ENSG00000171931	Q5XX13
ENSG00000173110	P17066	ENSG00000173157	P59510	ENSG00000173258	Q8TF39
ENSG00000175003	O15245	ENSG00000175197	P35638	ENSG00000175564	P55916
ENSG00000179148	Q9BYJ1	ENSG00000179388	Q06889	ENSG00000179520	Q8NDX2
ENSG00000179869	Q86UQ4	ENSG00000182175	Q96B86	ENSG00000182578	P07333
ENSG00000182866	P06239	ENSG00000183655	Q9H0H3	ENSG00000184205	Q9H2G4
ENSG00000185345	O60260	ENSG00000186517	Q7Z6I6	ENSG00000188089	Q3MJ16
ENSG00000189056	P78509	ENSG00000196569	P24043	ENSG00000197181	Q8TC59
ENSG00000197580	Q9BYV7	ENSG00000197632	P05120	ENSG00000198216	Q15878
ENSG00000198576	Q7LC44	ENSG00000198947	P11532	ENSG00000204103	Q9Y5Q3
ENSG00000204389	P0DMV8	ENSG00000205639	A6NFX1	ENSG00000211448	Q92813
ENSG00000211454	Q8NHP1	ENSG00000225217	P48741	ENSG00000240038	P19961
ENSG00000255150	Q8N140	ENSG00000260314	P22897	ENSG00000262655	Q9HCB6
ENSG00000273559	Q9NXE8	ENSG00000273777	P31997		

Input	Ensembl Id	Input	Ensembl Id	Input	Ensembl Id
ENSG00000057657	ENSG00000057657	ENSG00000073756	ENSG00000073756	ENSG00000087074	ENSG00000087074
ENSG00000087237	ENSG00000087237	ENSG00000100292	ENSG00000100292	ENSG00000102760	ENSG00000102760
ENSG00000105227	ENSG00000105227	ENSG00000106211	ENSG00000106211	ENSG00000108821	ENSG00000108821
ENSG00000115738	ENSG00000115738	ENSG00000116329	ENSG00000116329	ENSG00000118515	ENSG00000118515
ENSG00000119508	ENSG00000119508	ENSG00000120438	ENSG00000120438	ENSG00000120694	ENSG00000120694
ENSG00000120738	ENSG00000120738	ENSG00000122877	ENSG00000122877	ENSG00000124216	ENSG00000124216
ENSG00000128604	ENSG00000128604	ENSG00000130203	ENSG00000130203	ENSG00000130766	ENSG00000130766
ENSG00000132002	ENSG00000132002	ENSG00000132510	ENSG00000132510	ENSG00000134321	ENSG00000134321
ENSG00000138379	ENSG00000138379	ENSG00000144381	ENSG00000144381	ENSG00000149257	ENSG00000149257
ENSG00000149968	ENSG00000149968	ENSG00000159388	ENSG00000159388	ENSG00000160570	ENSG00000160570
ENSG00000162772	ENSG00000162772	ENSG00000164093	ENSG00000164093	ENSG00000164683	ENSG00000164683
ENSG00000164949	ENSG00000164949	ENSG00000166592	ENSG00000166592	ENSG00000167281	ENSG00000167281
ENSG00000168646	ENSG00000168646	ENSG00000169896	ENSG00000169896	ENSG00000170345	ENSG00000170345
ENSG00000171094	ENSG00000171094	ENSG00000173110	ENSG00000173110	ENSG00000175197	ENSG00000175197
ENSG00000182578	ENSG00000182578	ENSG00000184205	ENSG00000184205	ENSG00000197632	ENSG00000197632
ENSG00000198576	ENSG00000198576	ENSG00000204389	ENSG00000204389		

Interactors (3)

Input	UniProt Id	Interacts with	Input	UniProt Id	Interacts with
ENSG00000073756	EBI-5590670	P59595	ENSG00000168646	EBI-5237192	P35222
ENSG00000170345	EBI-9825772	P40763			

7. Identifiers not found

These 315 identifiers were not found neither mapped to any entity in Reactome.

ENSG00000006788 ENSG00000010295 ENSG00000013441 ENSG00000053524 ENSG00000076826 ENSG00000079393 ENSG00000080007 ENSG00000083896
ENSG00000086570 ENSG00000087589 ENSG00000088340 ENSG00000090512 ENSG00000091010 ENSG00000092345 ENSG00000099860 ENSG00000100095
ENSG00000100867 ENSG00000100987 ENSG00000104804 ENSG00000105146 ENSG00000105321 ENSG00000107742 ENSG00000107864 ENSG00000108551
ENSG00000109771 ENSG00000109794 ENSG00000110427 ENSG00000111011 ENSG00000111834 ENSG00000114529 ENSG00000114670 ENSG00000116299
ENSG00000116885 ENSG00000120820 ENSG00000121101 ENSG00000121297 ENSG00000122477 ENSG00000122733 ENSG00000124194 ENSG00000124251
ENSG00000125409 ENSG00000127743 ENSG00000128253 ENSG00000129654 ENSG00000130222 ENSG00000130477 ENSG00000132204 ENSG00000132952
ENSG00000134198 ENSG00000134940 ENSG00000137270 ENSG00000137960 ENSG00000138670 ENSG00000138769 ENSG00000140450 ENSG00000142621
ENSG00000143107 ENSG00000144655 ENSG00000146453 ENSG00000146592 ENSG00000147174 ENSG00000149646 ENSG00000149651 ENSG00000150051
ENSG00000152969 ENSG00000153303 ENSG00000153930 ENSG00000154099 ENSG00000157927 ENSG00000158023 ENSG00000158352 ENSG00000158486
ENSG00000160117 ENSG00000160781 ENSG00000162783 ENSG00000162944 ENSG00000162998 ENSG00000164287 ENSG00000164694 ENSG00000164744
ENSG00000164778 ENSG00000166016 ENSG00000166341 ENSG00000166492 ENSG00000166535 ENSG00000167476 ENSG00000167525 ENSG00000171540
ENSG00000171786 ENSG00000172578 ENSG00000172716 ENSG00000173545 ENSG00000174226 ENSG00000174792 ENSG00000174827 ENSG00000175213
ENSG00000175707 ENSG00000175985 ENSG00000176678 ENSG00000176761 ENSG00000176988 ENSG00000177453 ENSG00000177614 ENSG00000178297
ENSG00000178381 ENSG00000178440 ENSG00000179046 ENSG00000179314 ENSG00000179886 ENSG00000180336 ENSG00000181524 ENSG00000182308
ENSG00000182459 ENSG00000182518 ENSG00000183378 ENSG00000183496 ENSG00000183615 ENSG00000184368 ENSG00000185482 ENSG00000185633
ENSG00000185972 ENSG00000186364 ENSG00000187699 ENSG00000188039 ENSG00000188761 ENSG00000188984 ENSG00000189030 ENSG00000196900
ENSG00000197653 ENSG00000197748 ENSG00000197847 ENSG00000197980 ENSG00000198673 ENSG00000199879 ENSG00000204666 ENSG00000204929
ENSG00000207336 ENSG00000213013 ENSG00000213693 ENSG00000213892 ENSG00000213943 ENSG00000214107 ENSG00000214226 ENSG00000217527
ENSG00000218018 ENSG00000223356 ENSG00000224079 ENSG00000224280 ENSG00000224316 ENSG00000224429 ENSG00000225611 ENSG00000225808
ENSG00000226622 ENSG00000226891 ENSG00000228175 ENSG00000228242 ENSG00000228393 ENSG00000228509 ENSG00000228742 ENSG00000229419
ENSG00000229808 ENSG00000230453 ENSG00000230702 ENSG00000230825 ENSG00000231154 ENSG00000231890 ENSG00000232656 ENSG00000232811
ENSG00000233117 ENSG00000233143 ENSG00000233196 ENSG00000233290 ENSG00000233672 ENSG00000234498 ENSG00000235016 ENSG00000235082
ENSG00000235677 ENSG00000235823 ENSG00000236213 ENSG00000236480 ENSG00000236801 ENSG00000236882 ENSG00000237693 ENSG00000238121
ENSG00000240006 ENSG00000240219 ENSG00000241135 ENSG00000241322 ENSG00000243629 ENSG00000243648 ENSG00000243742 ENSG00000244040
ENSG00000244513 ENSG00000244682 ENSG00000245598 ENSG00000246203 ENSG00000246560 ENSG00000247199 ENSG00000247746 ENSG00000247982
ENSG00000249249 ENSG00000249550 ENSG00000249669 ENSG00000249715 ENSG00000249917 ENSG00000250240 ENSG00000250583 ENSG00000250848
ENSG00000251391 ENSG00000251537 ENSG00000253880 ENSG00000254473 ENSG00000254867 ENSG00000255171 ENSG00000255363 ENSG00000255508
ENSG00000255815 ENSG00000255931 ENSG00000255946 ENSG00000256206 ENSG00000256591 ENSG00000258359 ENSG00000258441 ENSG00000258469
ENSG00000258609 ENSG00000258667 ENSG00000258926 ENSG00000259463 ENSG00000259751 ENSG00000260448 ENSG00000260633 ENSG00000260641
ENSG00000260774 ENSG00000260799 ENSG00000261040 ENSG00000261094 ENSG00000261096 ENSG00000261654 ENSG00000261668 ENSG00000261684
ENSG00000264230 ENSG00000264490 ENSG00000266921 ENSG00000267136 ENSG00000267254 ENSG00000267270 ENSG00000267649 ENSG00000268006
ENSG00000268047 ENSG00000268223 ENSG00000268350 ENSG00000269091 ENSG00000269343 ENSG00000269720 ENSG00000270011 ENSG00000270607
ENSG00000270757 ENSG00000271862 ENSG00000272316 ENSG00000272630 ENSG00000273010 ENSG00000273373 ENSG00000275106 ENSG00000275437
ENSG00000277149 ENSG00000277151 ENSG00000277481 ENSG00000277498 ENSG00000277692 ENSG00000277957 ENSG00000278000 ENSG00000278771
ENSG00000279066 ENSG00000279103 ENSG00000279145 ENSG00000279456 ENSG00000279520 ENSG00000279529 ENSG00000279608 ENSG00000279641
ENSG00000279691 ENSG00000280012 ENSG00000282855 ENSG00000283209 ENSG00000283654 ENSG00000284977 ENSG00000285230 ENSG00000285278
ENSG00000285569 ENSG00000286235 ENSG00000286285 ENSG00000286782 ENSG00000286904 ENSG00000287007 ENSG00000287038 ENSG00000287054
ENSG00000287100 ENSG00000287217 ENSG00000287316 ENSG00000287437 ENSG00000287538 ENSG00000287853 ENSG00000287960 ENSG00000288000
ENSG00000288640 ENSG00000288852 ENSG00000288928 ENSG00000288995 ENSG00000289286 ENSG00000289486 ENSG00000289688 ENSG00000290058
ENSG00000290603 ENSG00000290705 ENSG00000291005
